# Diffusible signal factor signaling controls bioleaching activity and niche protection in the acidophilic, mineral-oxidizing leptospirilli

**DOI:** 10.1038/s41598-021-95324-9

**Published:** 2021-08-11

**Authors:** Sören Bellenberg, Beatriz Salas, Suresh Ganji, Cristian Jorquera-Román, Maria Luisa Valenzuela, Antoine Buetti-Dinh, C. Rikard Unelius, Mark Dopson, Mario Vera

**Affiliations:** 1 Centre for Ecology and Evolution in Microbial Model Systems (EEMiS), Linnaeus University, Kalmar, Sweden; 2 Department of Hydraulic and Environmental Engineering, Pontificia Universidad Católica de Chile, Av. Vicuña Mackenna 4860, Macul, 7820486 Santiago, Chile; 3 Department of Chemistry and Biomedical Sciences, Linnaeus University, Kalmar, Sweden; 4 Institute for Biological and Medical Engineering, Schools of Engineering, Medicine and Biological Sciences, Pontificia Universidad Católica de Chile, Av. Vicuña Mackenna 4860, Macul, 7820486 Santiago, Chile; 5 Grupo de Investigación en Energía y Procesos Sustentables, Facultad de Ingeniería, Instituto de Ciencias Químicas Aplicadas, Universidad Autónoma de Chile, Av. El Llano Subercaseaux 2801, San Miguel, Santiago de Chile, Chile; 6 Laboratory of Applied Microbiology (LMA), Department of Environment, Constructions and Design (DACD), University of Applied Sciences of Southern Switzerland (SUPSI), Via Mirasole 22a, 6500 Bellinzona, Switzerland; 7 Swiss Institute of Bioinformatics, Quartier Sorge – Batiment Genopode, 1015 Lausanne, Switzerland

**Keywords:** Applied microbiology, Biofilms

## Abstract

Bioleaching of metal sulfide ores involves acidophilic microbes that catalyze the chemical dissolution of the metal sulfide bond that is enhanced by attached and planktonic cell mediated oxidation of iron(II)-ions and inorganic sulfur compounds. *Leptospirillum* spp. often predominate in sulfide mineral-containing environments, including bioheaps for copper recovery from chalcopyrite, as they are effective primary mineral colonizers and oxidize iron(II)-ions efficiently. In this study, we demonstrated a functional diffusible signal factor interspecies quorum sensing signaling mechanism in *Leptospirillum ferriphilum* and *Leptospirillum ferrooxidans* that produces (Z)-11-methyl-2-dodecenoic acid when grown with pyrite as energy source. In addition, pure diffusible signal factor and extracts from supernatants of pyrite grown *Leptospirillum *spp*.* inhibited biological iron oxidation in various species, and that pyrite grown *Leptospirillum* cells were less affected than iron grown cells to self inhibition. Finally, transcriptional analyses for the inhibition of iron-grown *L. ferriphilum* cells due to diffusible signal factor was compared with the response to exposure of cells to N- acyl-homoserine-lactone type quorum sensing signal compounds. The data suggested that *Leptospirillum* spp. diffusible signal factor production is a strategy for niche protection and defense against other microbes and it is proposed that this may be exploited to inhibit unwanted acidophile species.

## Introduction

The industrial process of biomining describes the use of acidophilic microbes for the recovery of metals from sulfide ores. It includes bioleaching when the target metal is part of the metal sulfide mineral and biooxidation when the target metal is trapped as microscopic particles within the mineral matrix. Both processes are mediated by chemical dissolution of the metal sulfide bond that is enhanced by microbial oxidation of iron(II)-ions and inorganic sulfur compounds (ISCs). ISC oxidation to sulfuric acid contributes to the generation of an acidic medium while iron(II)-ion oxidation regenerates iron(III) ions, the chemical oxidant of the metal sulfide bond^1,2^. Acidophilic bacteria of the genera *Acidithiobacillus*, *Leptospirillum*, *Acidiphilium*, and *Sulfobacillus* are often present in sulfide mineral-containing environments between approximately 20–40 °C. For instance, *Leptospirillum* spp. often predominate in sulfide mineral-containing environments^3–6^ as they oxidize iron(II)-ions efficiently at high iron(III)/iron(II)-ion ratios^7,8^. Consequently, *Leptospirillum ferriphilum* often represents a significant portion of the microbial community in bioheaps for copper recovery from chalcopyrite (CuFeS_2_), the most abundant copper containing metal sulfide in the world^9^.

Cell attachment and biofilm formation on ores is mediated by extracellular polymeric substances (EPS) and is considered a critical process for mineral dissolution^2,10,11^. Attached cells on sulfidic ores significantly influence the dissolution kinetics^12,13^, and the degree of cell attachment to pyritic ores by iron oxidizing acidithiobacilli can be manipulated by pre-cultivation conditions, nutritional supplementation, pH, and ionic strength of the medium^14^. *Leptospirillum* species are efficient biofilm forming strains on pyrite and chalcopyrite^15^ due to high amounts of EPS embedding attached cells to the mineral surface^12,16,17^. However, it is unknown if other factors may influence mineral colonization by *Leptospirillum*, making them crucial for understanding cell attachment and biofilm formation on metal sulfides.

Quorum sensing (QS) comprises several types of cell–cell communication mechanisms mediated by the secretion of small ‘autoinducer’ molecules that regulate gene expression in a cell-density-dependent manner^18^. Acidophile LuxI/R type QS systems are present in several strains of *Acidithiobacillus ferrooxidans*, *Acidithiobacillus thiooxidans*, and *Acidiferrobacter *spp.^19,20^. In addition, synthetic N-acyl homoserine lactones (AHLs) modulate EPS production and biofilm formation in several acidophilic leaching bacteria^19–25^. However, *Leptospirillum ferrooxidans*, *L.* *ferriphilum*, and A*cidithiobacillus ferrivorans* lack AHL synthesis genes^20^. However they respond to external AHL addition, most likely due to the presence of orphan LuxR-like receptors^20,26^. Acidophiles also possess a cyclic diguanylate (c-di-GMP) signaling system that is synthesized in Gram-negative bacteria by several proteins with diguanylate cyclase activity (DGC) and degraded by phosphodiesterase (PDE) domain containing proteins. Intracellular c-di-GMP levels control several phenotypes such as chemotaxis, motility, EPS production, and biofilm formation^27^. QS and c-di-GMP are proposed to be the principal mechanisms regulating biofilm formation and EPS biosynthesis in Gram-negative acidophiles^28,29^. Furthermore, the presence of c-di-GMP metabolism in acidithiobacilli, *L. ferrooxidans*, and *Acidiferrobacter* sp. SPIII/3 and the increase of c-di-GMP levels measured in *At. ferrooxidans* ATCC 23270^T^ cells adhering to solid substrates suggests a connection between AHL mediated QS and the c-di-GMP pathways^20,24,26,30,31^. Another type of QS based system relies on diffusible signal factors (DSF) from a family of cis-2-unsaturated fatty acid signal compounds and DSF family signal sensing is known to act directly on c-di-GMP metabolism^32,33^. The main compounds identified are DSF ((Z)-11-methyl-2-dodecenoic acid) and *Burkholderia* diffusible signal factor (BDSF, (Z)-2-dodecenoic acid). Furthermore, DSF-signaling is associated with a strong inter-species and even inter-kingdom biofilm dispersal activity^32,34–37^. A complete DSF system is coded on the *L. ferriphilum* DSM 14647^T^ genome including DSF synthase (*rpfF,* LFTS_00514), a Hpt domain containing protein (LFTS_00515) adjacent to a signal transduction sensor kinase homologue (*rpfC*, LFTS_00516), and the respective two-component system response regulator encoding genes *rpfG* (LFTS_00517)^15,38^. DSF synthesis (RpfF) plus signaling and transduction (RpfC and RpfG) are mediated via the PDE HD-GYP domain of RpfG. Hence, lowered levels of c-di-GMP in the presence of DSF are thought to mediate biofilm dispersal and stimulate motility. Increased *rpf* gene RNA transcripts are present in continuous iron(II) cultures and in batch chalcopyrite cultures^39^ and the DSF synthase is enhanced in the planktonic population in axenic and mixed cultures^15^. *L. ferrooxidans* C2-3 exhibits a similar *rpf* gene cluster with a complete *rpfC* homologue^40^. In addition, the genomes of both *Leptospirillum* species encode multiple *rpfR* candidate genes. RpfR inhibits DSF synthase RpfF activity, unless DSF binds at RpfR PAS domain, causing conformational changes that release it from RpfF and trigger its PDE activity. A similar regulatory interaction with RpfF has been described for RpfC^32^. Consequently, we hypothesize that *Leptospirillum* species produce DSF-family signal molecules and that they are involved in regulating biofilm formation and bioleaching of metal sulfide ores.

Understanding microbial interactions with mineral surfaces along with inter- and intra-species cell-to-cell communication mechanisms are highly relevant for developing strategies to improve biomining. Recently, it was demonstrated that DSF and BDSF signal compounds have a strong inhibitory effect on the metabolic activity of bioleaching bacteria and their biofilm forming capabilities^15^. In this study, we test the hypothesis that DSF production by *Leptospirillum* spp. is a strategy for niche protection and defense against other bioleaching microbes and if this phenotype may be exploited in biomining to inhibit unwanted acidophile species.

## Results

### Presence of DSF-family compounds in cultures of *L. ferrooxidans* and *L. ferriphilum*

**Figure 1:**
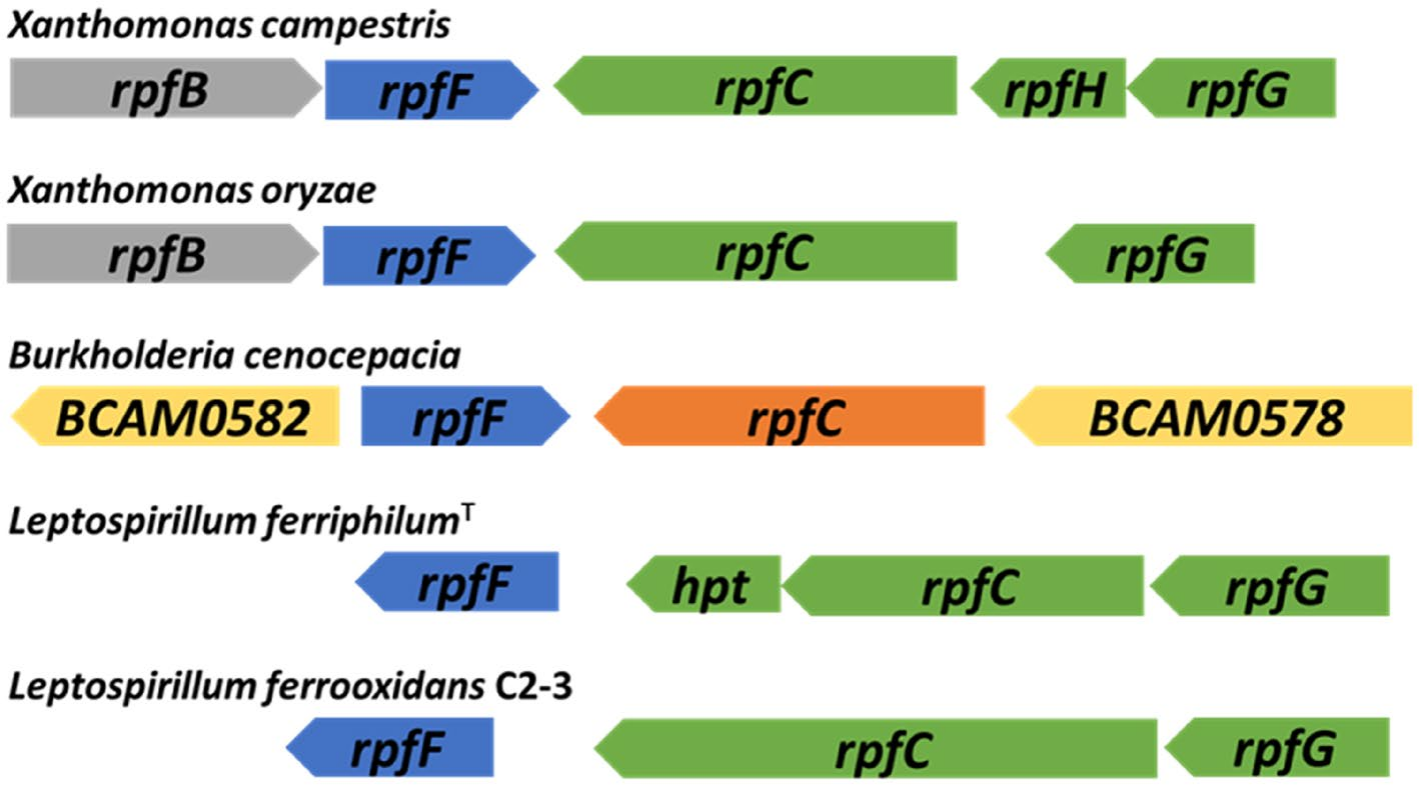
Organization of the *rpf* gene clusters in the genomes of *L.* *ferriphilum*^T^ and *L. ferrooxidans* C2-3. Gene clusters encoding the proteins of the DSF family QS systems have been compared with the model organisms *Burkholderia cenocepacia*, and two species of *Xanthomonas; X. campestris* and *X. oryzae*.

The organization of the *rpfFCG* gene clusters of both species is shown in Fig. 1. The DSF gene cluster of *L.* *ferriphilum* DSM 14647^T^ includes DSF synthase encoding gene *rpfF*, two genes encoding *rpfC* homologs. These are annotated as a “Hpt domain-containing protein”, and “signal transduction kinase”, encoding the receiver and the histidine phosphotranfer domains of RpfC and the phosphoacceptor domain histidine kinase A plus the histidine kinase like ATPase domain of RpfC, respectively. Also the two-component system response regulator-encoding gene *rpfG* is located in this gene cluster. *L.* *ferrooxidans* C2-3 exhibits a similar *rpf* gene cluster structure, with a complete *rpfC* homologue, encoding all the corresponding domains in the same annotated protein, located at a slightly longer distance from *rpfF* than other compared genomes. Other identified *rpf* homolog genes, in particular *rpfR* candidates, are encoded in different genomic locations, outside the *rpfFCG* cluster (Supplemental Fig S1). In *Xanthomonas campestris*, the genes encoding RpfC and RpfG are organized in an operon that is convergently transcribed to *rpfF*. This operon also contains *rpfH*, which encodes a protein similar to the input domain of RpfC, but with unknown function. The *rpfF* gene is found in an operon with *rpfB*, which encodes a fatty acyl-CoA ligase that may be involved in DSF processing. This organization of *rpf* genes occurs in all xanthomonads with the variation that *rpfH* is not widely conserved as seen for *Xanthomonas oryzae*. In *Burkholderia* species, the RpfFR system is widely conserved, where both genes *rpfF* and *rpfR* are convergently transcribed, whereas the genome of *B. cenocepacia* possess two flanking genes, *BCAM0227* and *BCAM0228*, which encodes an “accessory” system involved in BDSF signal transduction and response regulation, respectively. The flanking genes of DSF clusters in *L. ferriphilum* and *L. ferrooxidans* are unrelated, and showed no significant identity values to the known flanking genes of *Burkholderia* and *Xanthomonas*.

**Figure 2:**
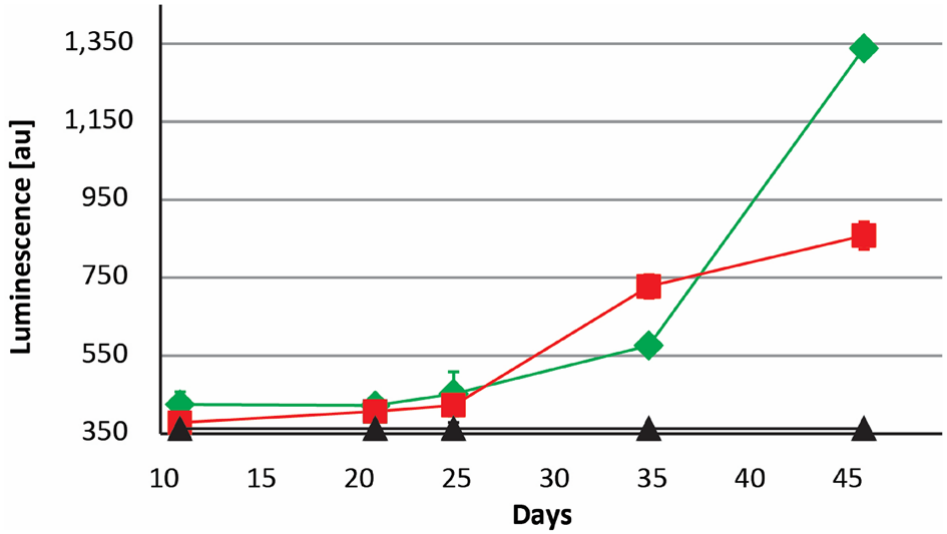
Bioassay for detection of BDSF and DSF in *Leptospirillium* spp. extracts cultured on pyrite. The black line and triangles indicate luminescence levels observed in control samples. The green line and diamonds or red line and boxes indicate luminescence levels in assays with extracts from *L.* *ferriphilum*^T^ or *L.* *ferrooxidans*^T^, respectively. Data are averages ± SD of luminescence reads from triplicate experiments (*n* = 3).

The biosensor strain *Burkholderia cenocepacia* H111-*rpfF*_*Bc*_ (pan-L15) was used to confirm that DSF and BDSF could be efficiently extracted from acidic culture medium in pyrite cultures using dichloromethane (Supplemental Fig. S2). As previously described^41^, the biosensor was more sensitive for detecting BDSF than DSF and a semi-quantitative estimation was possible for pure synthetic substances (Supplemental Fig. S3). DSF-family compounds were found in batch pyrite cultures of both *Leptospirillum* species, especially at later stages after 25 days of cultivation when an elevated luminescence suggested that DSF family molecules accumulated in pyrite culture supernatants (Fig. 2). Extracts from 500 mL stationary phase iron(II)-grown cultures did not trigger luminescence of the biosensor (not shown) while extracts from pyrite grown *L. ferriphilum* cells inhibited cell growth and iron oxidation in cultures of *Acidiferrobacter* SPIII/3 and *L.* *ferrooxidans*. (Supplemental Fig. S4).

### Analysis of DSF production by *L. ferrooxidans* and *L. ferriphilum*^T^

**Figure 3:**
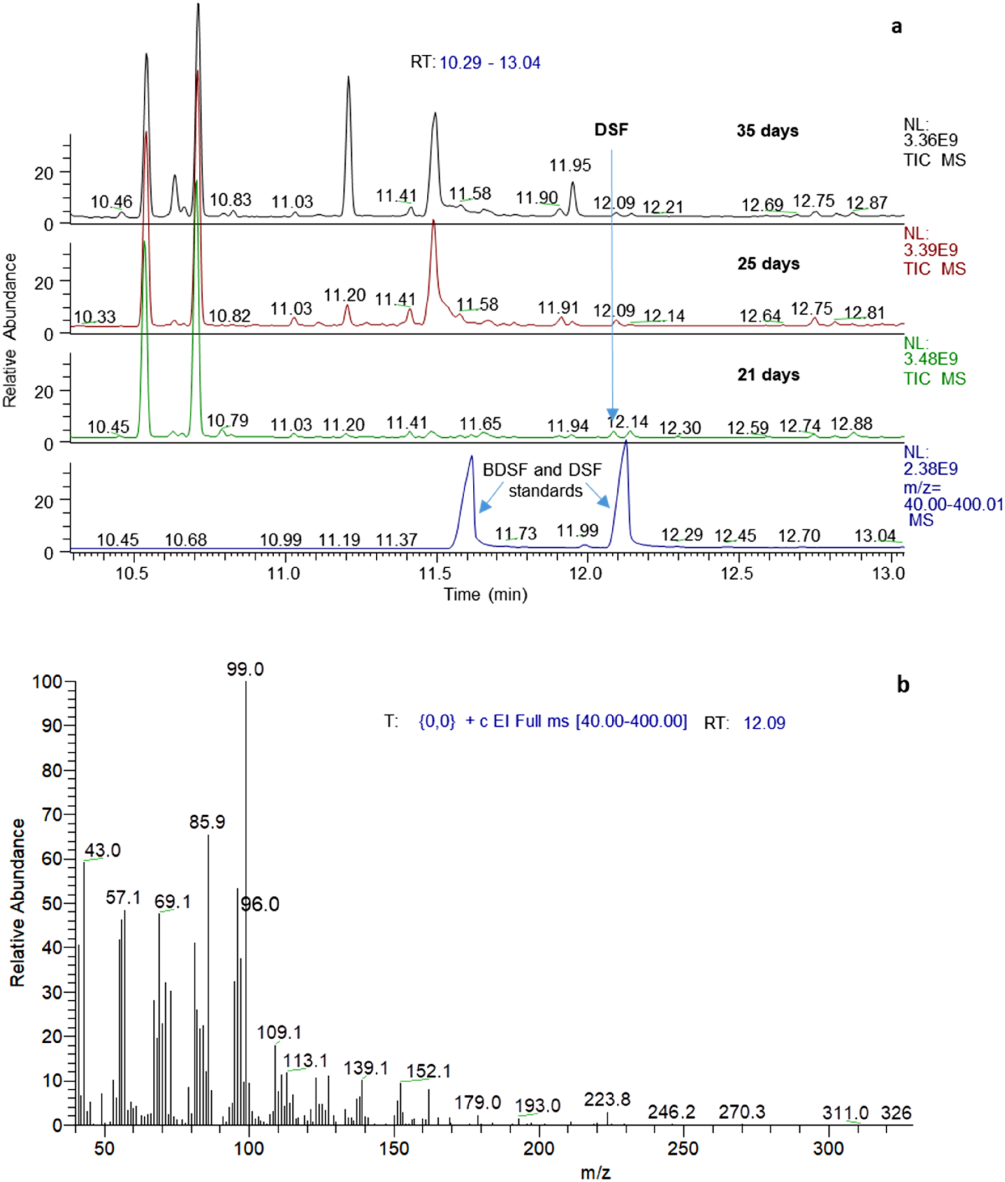

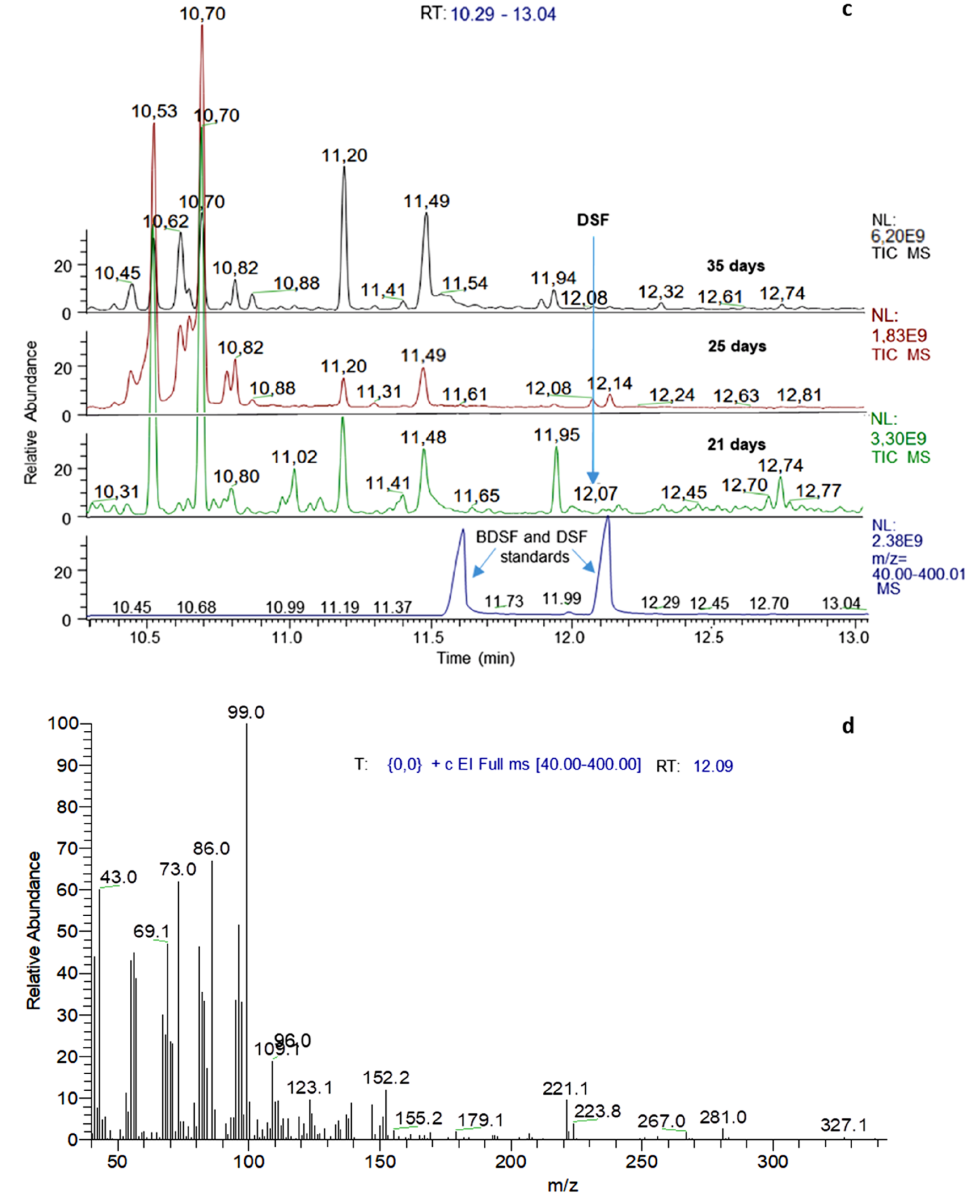
GC–MS analysis of culture extracts for identification of biogenic DSF. Total ion chromatograms of extracts from 21, 25, and 35 days old *L. ferrooxidans*^T^ (**a**) and *L.* *ferriphilum* DSM^T^ (**c**) pyrite cultures measured by GC–MS. The mass spectrum at the inferred retention time of DSF (12.09 min) confirmed the presence of the biogenic substance in pyrite culture supernatants of *L. ferrooxidans*^T^ (**b**) and *L.* *ferriphilum*^T^ (**d**).

**Figure 4:**
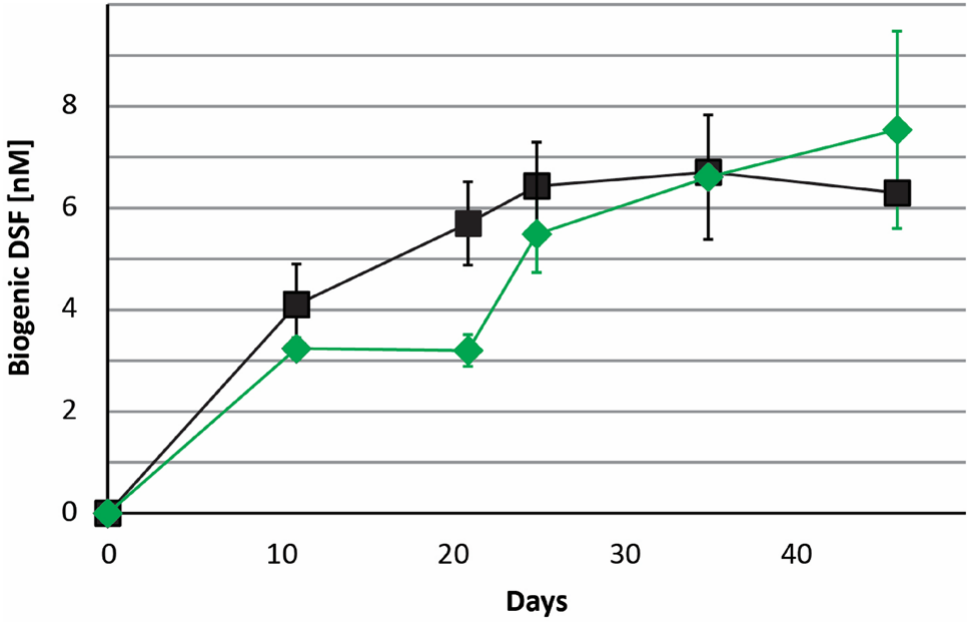
Development of DSF concentrations in *Leptospirillum* spp*.* culture supernatants. Concentration of biogenic DSF in cultures of *L. ferrooxidans*^T^ (black boxes) and *L.* *ferriphilum*^T^ (green diamonds) as calculated using GC–MS chromatogram peak areas. Data are averages ± SD from triplicate experiments (*n* = 3) using normalized peak areas (internal standard) converted to concentration levels using an external calibration.

Comparison of retention times of synthetic DSF in gas chromatography with peaks in culture extracts and the characteristic fragmentation patterns of these peaks in gas chromatography-mass spectrometry (GC–MS) unambiguously identified the presence of biogenic DSF in cultures of both *Leptospirillum* species (Fig. 3), although with differing development profiles (Fig. 4). Namely, the DSF concentration in *L. ferrooxidans* pyrite cultures increased from 4.1 ± 0.8 nM (all *n* = 3) on day 11 to a plateau at 6.4 ± 0.2 nM on day 25 and remained constant. In contrast, *L. ferriphilum*^T^ pyrite cultures had a low and constant DSF level at 3.2 ± 0.2 and 3.2 ± 0.3 nM on days 11 to 21 followed by an increase to 6.6 ± 1.2 nM on day 35 and 7.5 ± 1.9 nM on day 46.

### Physiological effects of synthetic DSF and BDSF on growth and iron oxidation

**Figure 5:**
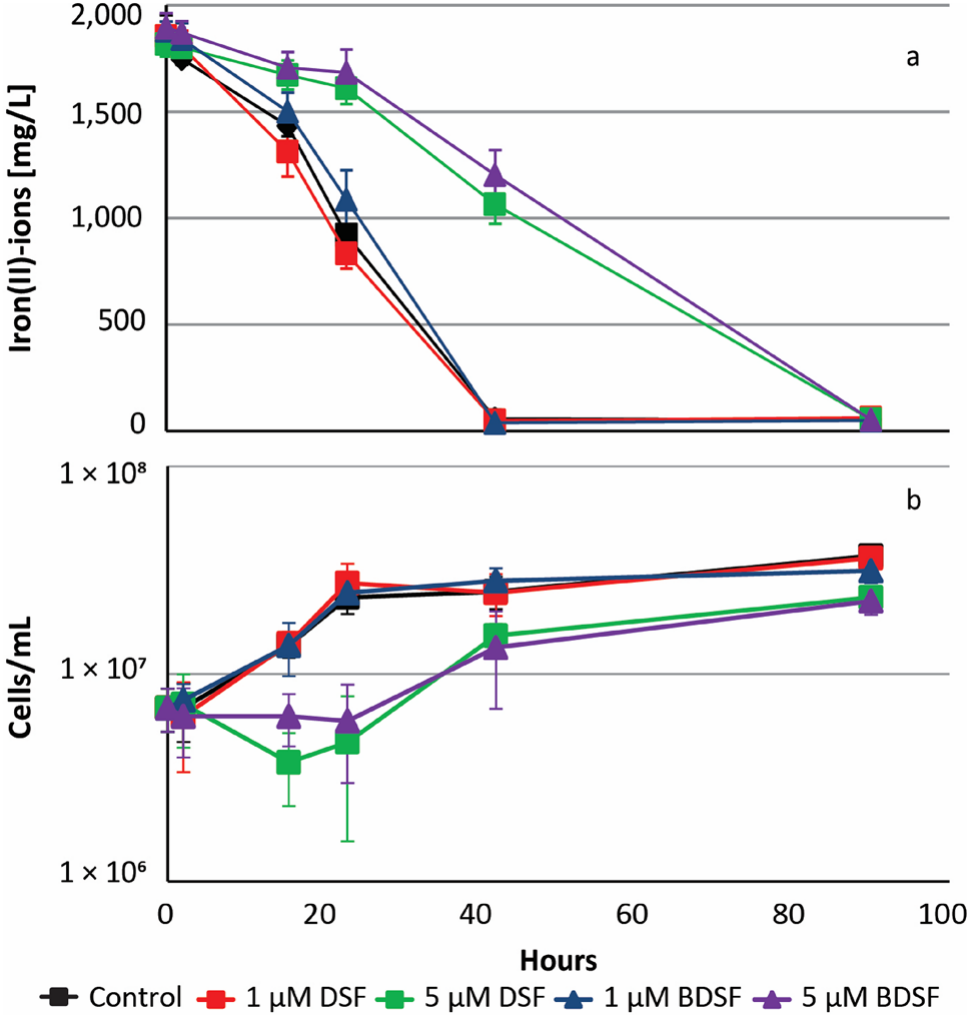
DSF or BDSF inhibit biological iron oxidation and cell growth. Cultures were inoculated with 10^7^ cells/mL iron(II)-grown cells of *L.* *ferriphilum*^T^. DSF or BDSF was added at 1 or 5 µM 16 h before 32 mM iron(II)-ions were added. Cells are inhibited in iron(II) oxidation (**a**) and growth (**b**) in presence of 5 µM DSF ((Z)-11-methyl-2-dodecenoic acid, green boxes) or 5 µM BDSF ((Z)*-*2-dodecenoic acid, purple triangles).

Iron(II)-grown cells of all the strains used in this study were inhibited regarding their ability to oxidize iron(II)-ions by exposure to 5 µM DSF or BDSF. For instance, *L. ferriphilum*^T^ iron(II) oxidation was delayed after exposure to 5 µM DSF or BDSF (Fig. 5) and once the inhibition was overcome, the rate of iron oxidation was significantly lower (Welch t-test, *p* = 0.0029) at 0.40 ± 0.03 mmol/h as compared to the control assay or after exposure to 1 µM effector compounds (0.91 ± 0.08 mmol/h). In consequence, both the growth rate and cell yield were significantly lower after exposure to 5 µM DSF or BDSF (Fig. 5b).

**Figure 6:**
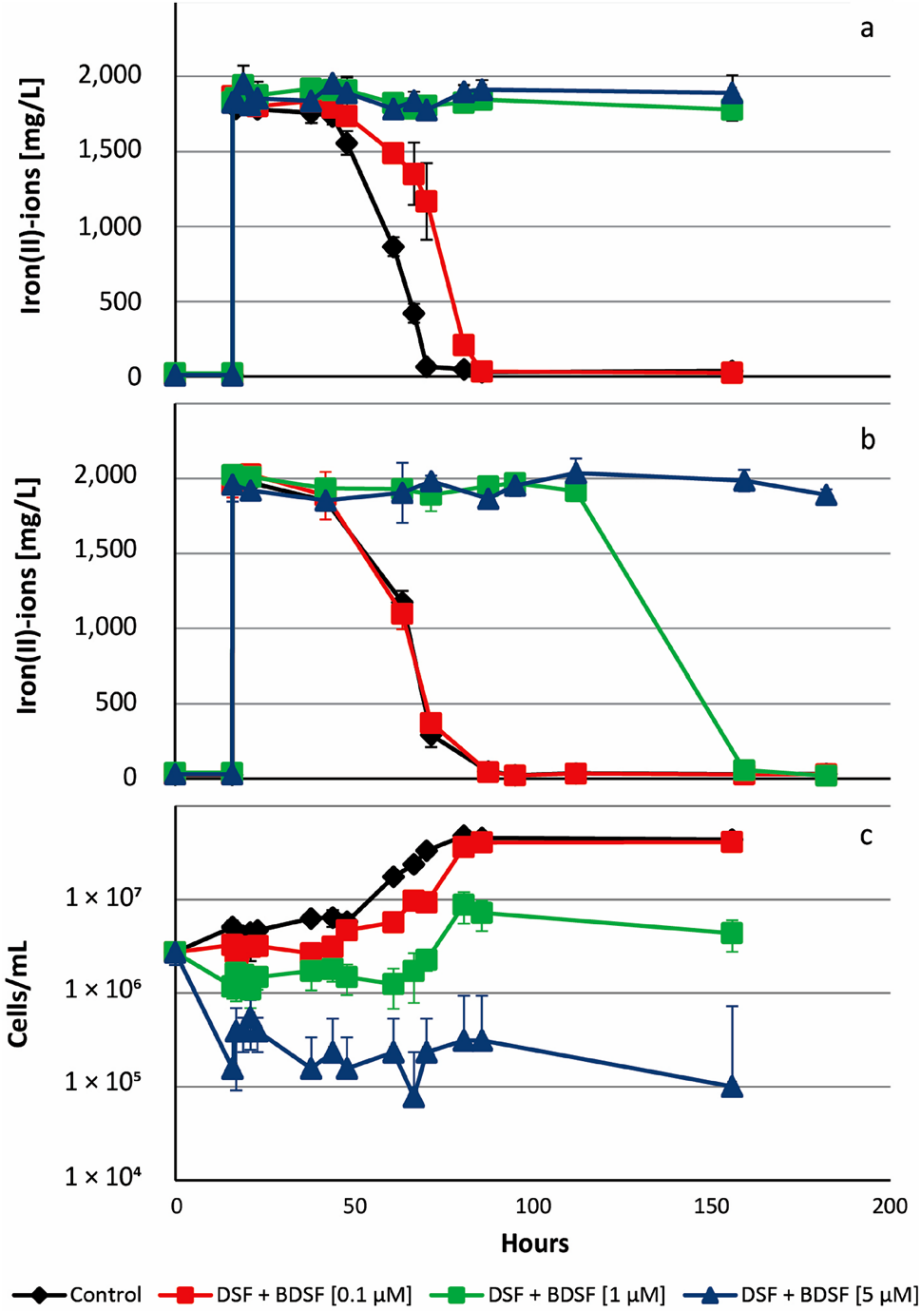
Iron-grown cells of *L.* *ferriphilum*^T^ are more sensitive to inhibition by DSF molecules than pyrite grown cells. Cultures were inoculated with 4 × 10^6^ cells/mL cells of *L. ferriphilum*^T^ and exposed to 0.1, 1, and 5 µM DSF ((Z)-11-methyl-2-dodecenoic acid) and BDSF ((Z)-2-dodecenoic acid) for 16 h before 32 mM iron(II)-ions were added. Iron(II)-grown (**a**) and pyrite-grown cells (**b**) were differentially inhibited in iron(II) oxidation. Growth in presence of increasing concentrations DSF/BDSF is strongly effected as shown for iron(II)-grown cells (**c**).

DSF and BDSF were also tested in combination at 0.1, 1, and 5 µM per compound that resulted in combined concentrations of 0.2, 2, and 10 µM DSF-family compounds (Fig. 6). The effects of 10 µM addition were more pronounced (e.g. an initial cell lysis; Fig. 6c) when using a lower inoculum size (4 × 10^6^ cells/mL) and hence, a higher relative concentration of DSF or BDSF per cell. Cell growth in assays with iron(II)-grown cells followed the trend of iron oxidation. Delayed iron(II) oxidation was observed with 0.2 µM, while iron oxidation did not occur within 150 h at 2 and 10 µM. Iron(II)-grown cells of *L. ferriphilum* were more sensitive to the presence of DSF and BDSF than pyrite-grown cells. For instance, the latter were unaffected in presence of 0.2 µM DSF family compound and iron oxidation was observed 100 h after inoculation in assays with 2 µM.

**Figure 7:**
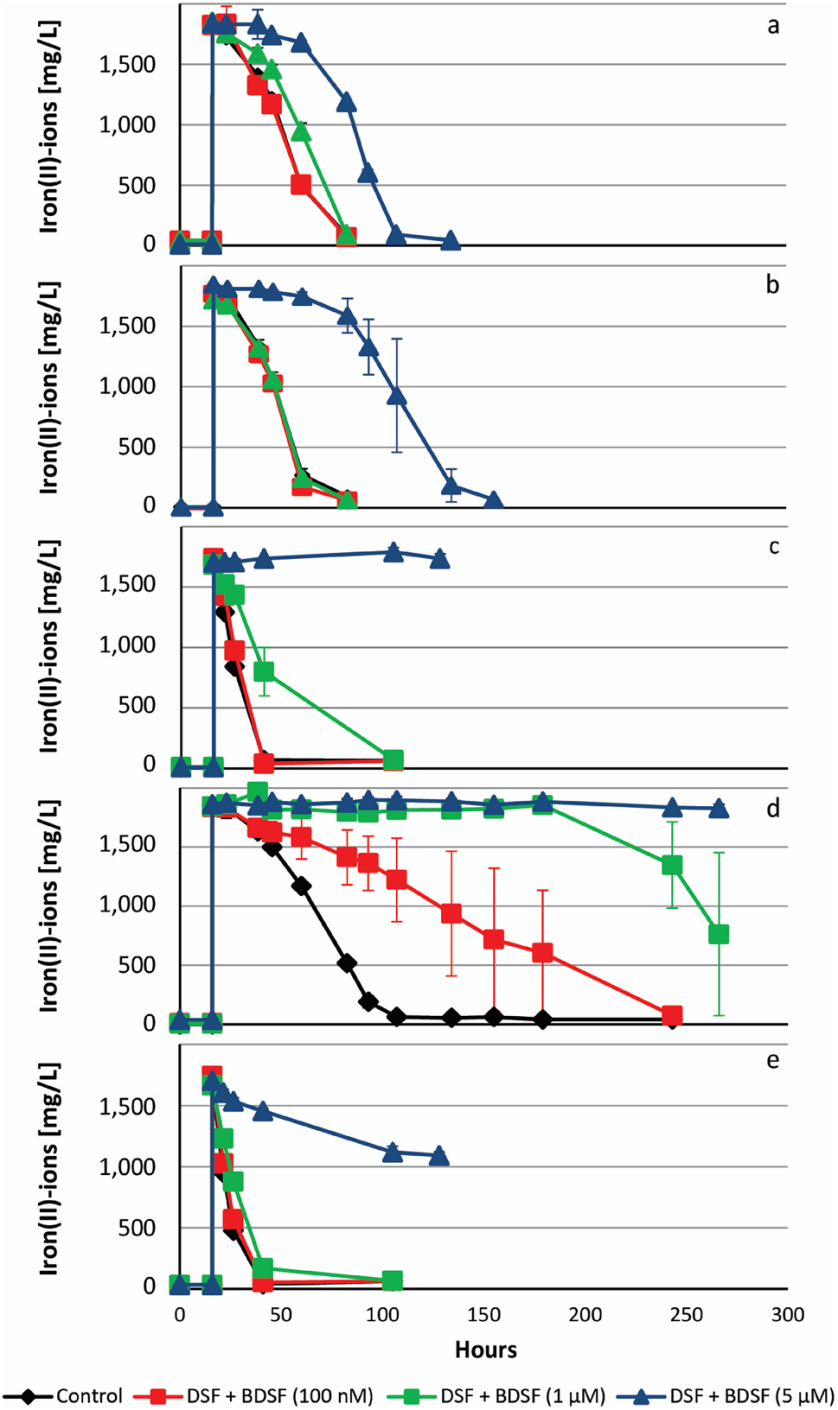
DSF and BDSF inhibit biological iron oxidation in various acidophilic bacteria. Cultures were inoculated with 10^7^ cells/mL iron(II)-grown cells of *At. ferrooxidans* ATCC 53,993 (**a**), *At. ferridurans*^T^ (**b**), *L. ferrooxidans*^T^ (**c**), *Acidiferrobacter* sp. SPIII/3 DSM 27,195 (**d**), and *S. thermosulfidooxidans*^T^ (**e**) and exposed to DSF ((Z)-11-methyl-2-dodecenoic acid) and BDSF ((Z)-2-dodecenoic acid) for 16 h before 32 mM iron(II)-ions were added.

Similar qualitative effects regarding cell growth (not shown) and iron oxidation were observed with iron(II)-grown cells of *L.* *ferrooxidans*^T^ and with members of other bacterial phyla, such as *At. ferrooxidans* ATCC 53993, *Acidithiobacillus ferridurans* ATCC 33020^ T^, *Acidiferrobacter* sp. SPIII/3 DSM 27195, and *Sulfobacillus thermosulfidooxidans* DSM 9293^T^ (Fig. 7).

### Effect of DSF-family and AHL- signal compounds on iron(II)-grown *L. ferriphilum*^T^ RNA transcripts

**Figure 8:**
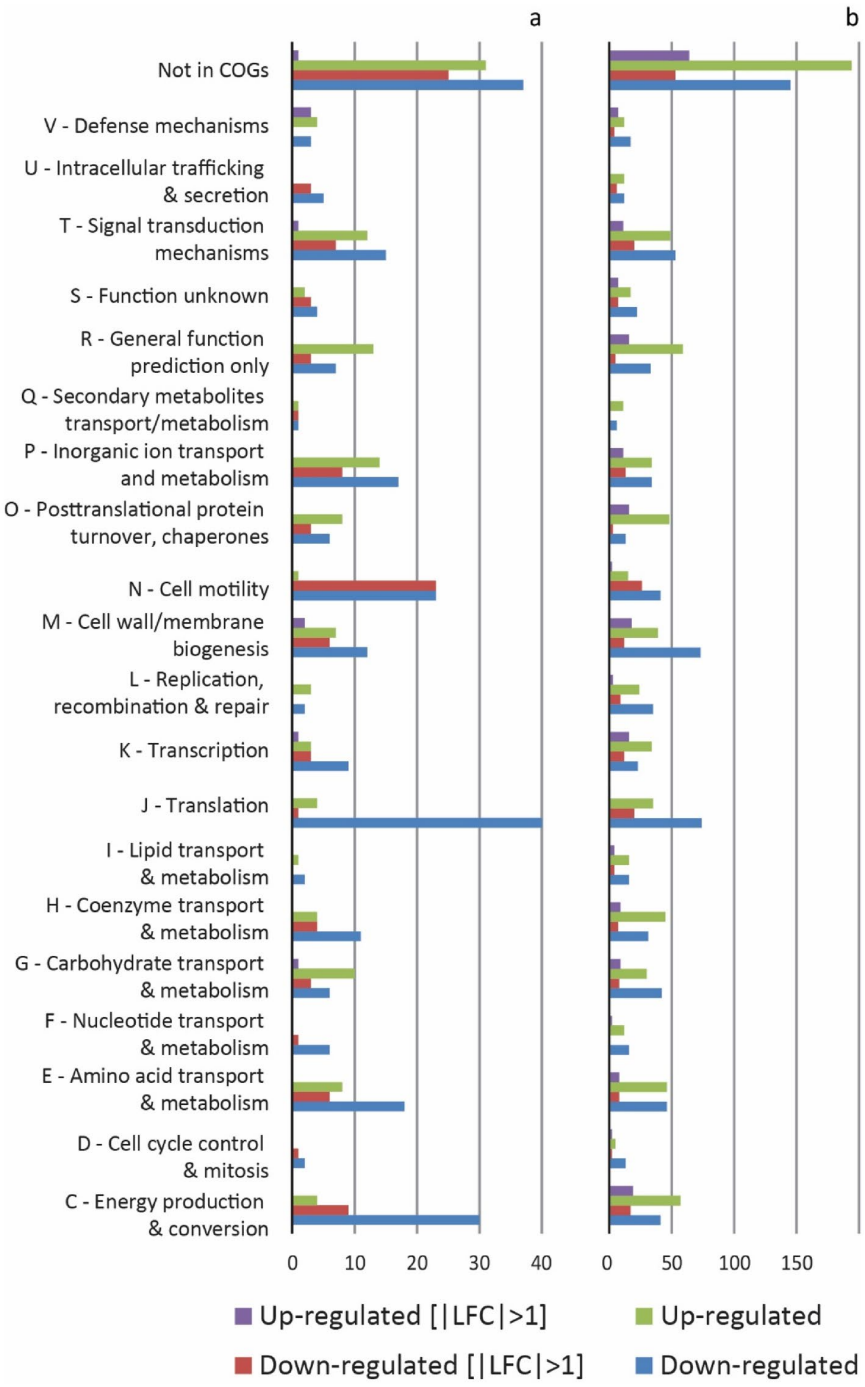
Clusters of orthologous groups (COGs) analysis of transcriptional changes in cells of *L. ferriphilum* DSM 14647^ T^ after exposure to DSF/BDSF (**a**) and AHLs **(b).** Numbers COG-assigned differentially expressed genes with *p*adj < 0.005 are shown as green and blue bars for higher and fewer RNA transcripts, respectively (393 and 1450 genes in total for DSF/BDSF and AHLs, respectively). Purple and red bars indicate the fraction of RNA transcripts with |LFC|> 1 (100 and 419 genes for DSF/BDSF and AHLs, respectively).

Analysis of RNA transcripts after exposure of iron(II)-grown cells of *L.* *ferriphilum*^T^ to DSF and BDSF revealed 100 genes with significantly differing transcript numbers (|Log_2_-fold change (LFC)|> 1, *p* value < 0.05; Supplemental Table S1). The majority (92 of 100) of these genes were significantly decreased and were associated with respiration, central carbon metabolism, biosynthesis of amino acids, iron-sulfur clusters, cytochromes, nucleotides, vitamins, lipopolysaccharides, cell walls, and flagella (Supplemental Table S1). Six of the eight genes with increased RNA transcript counts when exposed to DSF/BDSF encoded proteins were annotated as components of multidrug efflux systems (LFTS_00939 -_00941, _02071 - _02073). The toxic effect of DSF/BDSF was also supported by the functional annotation of those genes by COG analysis (Fig. 8a). Three of those genes with increased transcripts were categorized in COG V (defense mechanisms; LFTS_00941, _02072, _02073), two were multidrug-export membrane proteins in COG M (cell wall/membrane synthesis, LFTS_02071, _02073), and one was a related transcriptional regulator COG K (transcription, LFTS_00939), located within the putative operon structure of one of the multidrug efflux systems (LFTS_00939 -_00941). The remaining two genes (LFTS_01634 and _01845) were annotated as trehalose 6-phosphate phosphatase (*otsB*) and diguanylate cyclase (GGDEF) domain-containing protein (*dosC*), respectively.

**Figure 9:**
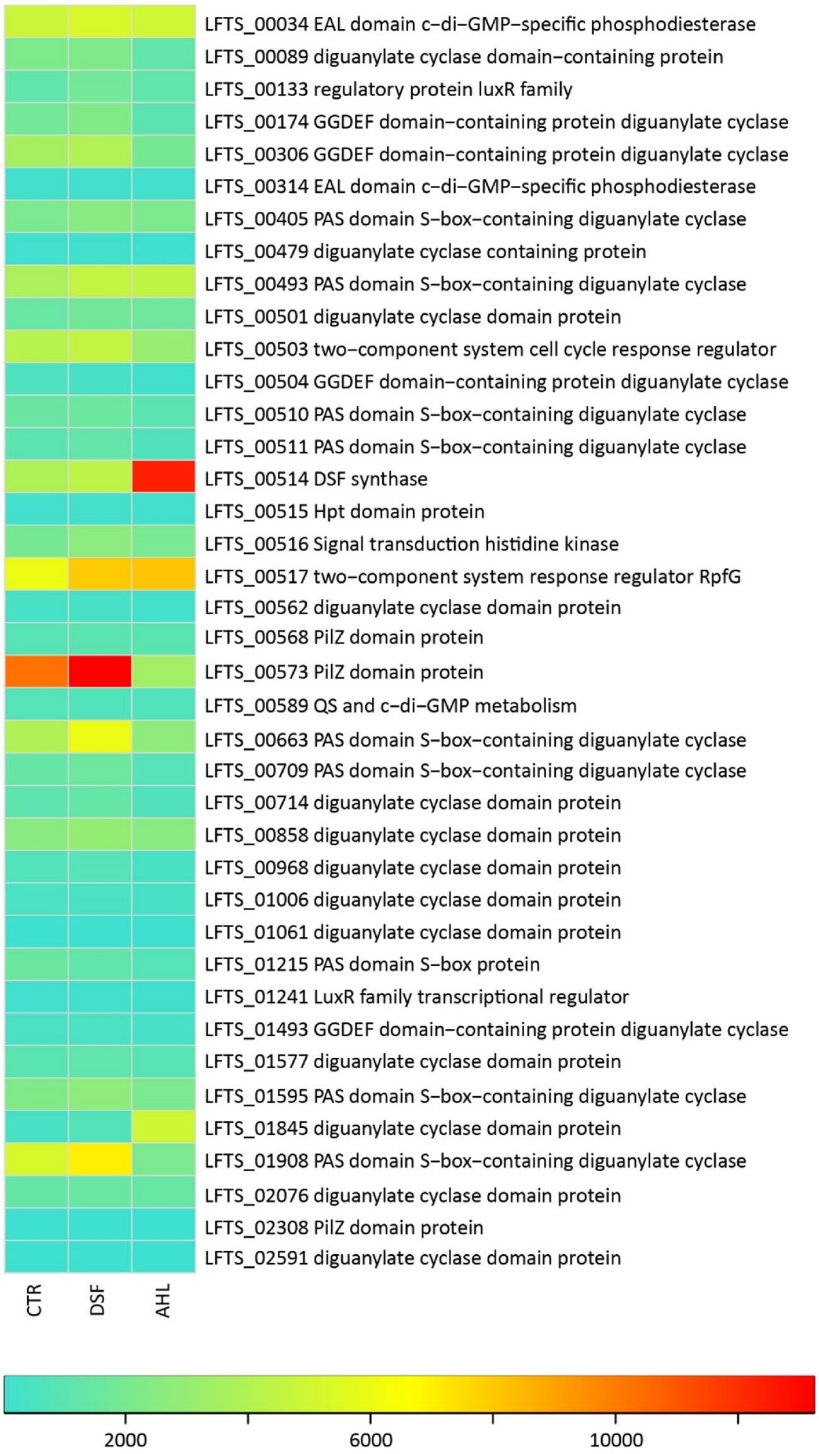
Heatmap of averaged counts of transcripts of QS and c-di-GMP metabolism genes. Color-coded averaged counts of transcripts indicate low (0; turquoise), medium (6500; yellow) and high (13,000; red) counts of gene transcripts in RNA samples from cells exposed to DSF molecules (DSF), N-acyl-homoserine-lactones (AHL) or control cells (CTR).

Analysis of the transcriptomes after two hours of treatment with the AHL mixture resulted in 419 statistically different expressed transcripts (|LFC|> 1, *p* value < 0.05) of which 206 and 213 transcripts were increased and decreased, respectively (Supplemental Table S2 shows a sub-set of transcripts with |LFC|> 2, *p* value < 0.005). Transcripts with decreased counts included genes in the COG categories translation (J), intracellular trafficking and secretion (U), signal transduction (T), and cell motility (N), respectively (Fig. 8b). Transcripts with increased counts included categories R (general function prediction only), M (cell wall/membrane biogenesis), O (posttranslational modification, protein turnover, chaperones), and V (defense mechanisms) as well as unassigned genes not in COGs. More specifically, *rpf*F (DSF-synthase, LFTS_00514) transcript counts were increased in response to exposure to AHLs (Fig. 9 and Supplemental Table S2). In general, the transcriptomes indicated low transcript counts of the sensor Hpt domain containing protein encoding gene (LFTS_00515) while the average transcript counts of the adjacent *rpfC* sensor kinase (LFTS_0516) were one order of magnitude higher. Both genes had slightly increased transcripts in cells exposed to DSF/BDSF and AHLs (LFTS_00515; DSF: LFC = 0.53 and *p* = 6 × 10^–3^, AHL: LFC = 0.69 and *p* = 10^–3^; LFTS_0516; DSF: LFC = 0.38 and *p* = 4 × 10^–4^, AHL: LFC = 0.54 and *p* 2 × 10^–9^). Likewise, transcripts coding for the cognate response regulator *rpf*G (LFTS_00517) were increased in cells exposed to DSF/BDSF or AHLs (LFTS_00517; DSF: LFC = 0.29 and *p* = 4 × 10^–4^, AHL: LFC = 0.87 and *p* = 3 × 10^–34^). Furthermore, AHL-treatment caused increased transcript counts for the same multidrug efflux systems as for DSF/BDSF treatment plus one additional multidrug efflux system (LFTS_00325 - _00327, _00329). However, even though these defense systems were triggered, toxic effects of AHLs or inhibition of iron oxidation as compared to treatment with comparable concentrations of DSF or BDSF (Figs. 6 and 7) were not observed. The active growth of *Leptospirillum* cells in the presence of AHLs was also reflected by several genes with increased transcript counts that were associated with functions such as carbon and nitrogen fixation, respiration, central carbon metabolism, ammonium uptake, biosynthesis of amino acids, iron-sulfur-clusters, cytochromes, nucleotides, vitamins, lipopolysaccharides, and cell walls/membranes. Finally, a further parallel finding after treatment with the AHL mixture and DSF/BDSF was lower transcript counts for flagella biosynthesis and chemotaxis related genes (Supplemental Table S2).

## Discussion

Biofilm communities are crucial in biomining since they regenerate iron(III)-ions that oxidize the metal sulfide directly on the mineral surface. However, too thick biofilms may passivate the mineral surface due to limitation of rate determining iron-ion and ISC transport processes. Consequently, biofilm dispersal is important for the biologically catalyzed metal sulfide oxidation e.g. in heap bioleaching processes. This is by ensuring that mono-layer biofilms are dominant^11^ to support contact of metal sulfide surfaces with the leaching solution and facilitating colonization of downstream mineral surfaces. DSF-signaling has been widely reported as a biofilm dispersal factor in a broad range of species^32,34^. Recently we demonstrated this effect in some bioleaching bacteria, observing a decrease in mineral-attached *Acidithiobacillus caldus*, *L. ferriphilum*, and *S. thermosulfidooxidans* populations on mineral particles after DSF addition^15^. It was also proposed that biofilm dispersal upon external addition of DSF family signal compounds is an active biological function of DSF signaling in leptospirilli.

The other major effect of DSF is the inhibition of biological iron oxidation (Figs. 6, 7, 8), which causes inhibition of bioleaching and a concentration dependent toxic effect of DSF/BDSF was reflected in the transcriptomic analysis. Micromolar DSF/BDSF concentrations are unlikely to occur in leptospirilli containing habitats and the biogenic concentrations of DSF in batch pyrite cultures (Fig. 4) were one order of magnitude below the lowest inhibitory levels that were tested in the iron(II) oxidation assays. However, we suggest this phenomenon will be at least partially biologically relevant in close proximity to mineral attached *Leptospirillum* cells as elevated concentrations likely occur via fatty acid accumulation in cell membranes and the EPS layer. Pyrite-grown cells were shown to be adapted to the presence of biogenic DSF family compounds (Fig. 6), while unadapted intruding cells will be inhibited, preventing access to the energy source. Consequently, the previously described mutual inhibitory interaction in pyrite cultures inoculated with *L.* *ferrooxidans* and *Acidiferrobacter* sp. SPIII/3^20^ was likely a result of the high sensitivity of *Acidiferrobacter* sp. SPIII/3 to DSF/BDSF that inhibited growth or even caused cell lysis, while its AHL production or exudates potentially inhibited *L.* *ferrooxidans*. A second example of an interspecies interaction likely mediated via DSF was the decreased leaching performance by *S. thermosulfidooxidans* cells added to pyrite cultures pre-colonized by *L. ferriphilum* compared to simultaneous inoculation with *L. ferriphilum* and *S. thermosulfidooxidans*^42^. This was potentially due to DSF release by *L. ferriphilum* mineral-oxidizing micro-colonies prior to the addition of *S. thermosulfidooxidans*. DSF then specifically inhibits iron(II) oxidation in intruding *S. thermosulfidooxidans* cells. The effect might also contribute to the preference for ISC oxidation by *S. thermosulfidooxidans* when in co-culture with *L. ferriphilum*^43^, while in batch cultivation systems exudates from *S. thermosulfidooxidans* likely inhibit *L. ferriphilum*^T^. In this work, it was also shown that extracts from pyrite grown cultures of *L. ferriphilum*^T^ were able to inhibit *Acidiferrobacter* sp. SPIII/3 and *L. ferrooxidans* iron oxidation. Previously it has been shown that *L. ferriphilum*^T^ extracts widely inhibit iron oxidation of the Gram-positive *S. thermosulfidooxidans*^T^ and *Acidimicrobium ferrooxidans*, as well as in several acidithiobacilli, such as *At. ferrooxidans*, *At. ferrivorans* and *At. ferriphilus*. Interestingly, the addition of *L. ferriphillum*^T^ extracts showed no inhibitory effects against cultures of *S. thermosulfidooxidans*, *At. caldus*, and *At. thiooxidans* when grown using elemental sulfur as the sole energy source^44^. We propose that the production of DSF by actively mineral-oxidizing cells is a sound strategy to keep the mineral energy source primarily accessible to DSF-producing leptospirilli. Therefore, and taking into account that pyrite grown *L. ferriphilum*^T^ cells were less sensitive regarding inhibition of iron oxidation by DSF, it is likely that at the micro-colony scale the niche defense hypothesis explains the biological advantage of DSF production in leptospirilli. In this context, the production of DSF could also contribute to modulate substrate oxidation of competing neighbor species by switching-off their iron oxidation pathways, without interfering on their capacities to oxidize ISCs. In addition, that DSF-family signal compounds are biofilm dispersal factors is also in agreement with the observation that in a filtered pyrite leachate from *L. ferriphilum, S. thermosulfidooxidans* tend to oxidize ISCs instead of ferrous iron at decreased numbers of mineral-attached cells^42^. Bioleaching communities are complex, and several heterotrophic microorganisms are always present. These exert complex nutritional interactions with iron/sulfur oxidizing chemolithotrophs by feeding organic carbon and substrates that can be toxic for the chemolithotrophs^50^. In the future, it will be interesting to determine if there are effects related to the presence of DSF in mixed cultures containing *Leptospirillum*, *Acidithiobacillus* as well as heterotrophic species such as *Acidiphilium* spp.

DSF and BDSF inhibited iron oxidation to varying degrees in all tested iron-oxidizers (Fig. 7) raising the question of the inhibitory mechanism. Organic acids are protonated at pH values below their specific pK_a_ value and hence, DSF-family compounds are protonated in the experimental conditions and act as uncouplers by entering cells and releasing a proton within the near pH neutral periplasm. This readily explained elevated RNA transcript counts for multidrug efflux systems after DSF family compound addition. However, *At. ferrooxidans*, former *Thiobacillus ferrooxidans* strains have been shown to grow in the presence of small organic acids, such as acetic or citric acid, at concentrations three or four orders of magnitude higher than the inhibitory or lethal levels for DSF family compounds^45–48^. In addition, Aston et al., 2009 reported the toxic effects of several organic acids to *At. caldus*, strain BC13. This included oxaloacetate, pyruvate, acetate, malate, succinate, fumarate, oxaloacetate and 2-ketoglutarate. All of them exhibited toxicity between 1–5 mM, while oxaloacetate was toxic at 0.25 mM^49^. Nancucheo & Johnson, in 2010, reported toxicity of glycolic acid against *L. ferriphilum*^T^ and *L. ferrooxidans*^T^. The maximum concentration of glycolic acid that permitted growth was close to the mM range, with 0.5 and 0.1 mM, respectively^50^. Interestingly, the acyl-chain length of fatty acid correlates with enhanced toxicity^51^ and long-chain fatty acids are known for their detrimental effect on the performance of anaerobic sewage sludge digestion processes and their inhibitory effect on methanogenic archaea^52,53^. Furthermore, unsaturated fatty acids are known antimicrobials that cause membrane disruption in Gram-positive bacteria^54^. Therefore, the observed DSF transcriptional effect would not only involve QS signaling via DSF perception and response regulator components, but would also potentially include an acid stress response upon direct uptake of DSF molecules. This was supported by the transcriptional analyses, which showed *rpfF* transcript counts were not significantly increased upon DSF/BDSF treatment but were increased after AHL addition. Also, the DSF/BDSF effect could not be related to its sensing and response regulation via RpfC and RpfG proteins. Interestingly, *flrB* transcript levels were decreased in *L. ferriphilum* after both DSF/BDSF and AHL mixture treatments. In *Vibrio alginolyticus* it has been reported that *flr* genes (including *flrA*, *flrB*, and *flrC*) influence bacterial adhesion, motility, biofilm formation, and EPS production and some strains cultured under Cu^2+^, Pb^2+^, Hg^2+^ or low-pH conditions showed a significant down-regulation of these genes, leading to deficiencies in adhesion, motility, flagellar assembly, biofilm formation, and EPS production^55^. In agreement with these observations, DSF and AHL addition produced a decrease in transcript counts of *L. ferriphilum* flagellar related genes, flagellar basal-body rod proteins (*flg*), flagellar biosynthetic protein (*flh*), and other flagellar genes (*fli*). As DSF was added to planktonic cultures, the transcript decay observed for flagellar genes and motility functions suggested that cells switched to a sessile state. In contrast, natural DSF signaling in *X. campestris* pv. *campestris* and *Xanthomonas axonopodis* pv. *citri* increases the expression of genes involved in flagellum biosynthesis^56,57^. This difference may be due to the different experimental approaches along with the effects of gene deletions that could be more complex or unexpectedly influence other regulatory pathways, compared to external addition of signalling molecules. For example, in *Cronobacter turicensis* it has been reported that the flagellar regulon-associated gene *flhE* is unaltered in the *rpfF*/*R* mutants, but it is significantly upregulated in complemented mutants carrying additional copies of these genes^58^. We also highlight that the organization of the *rpfFCG* gene cluster as a putative uni-directional operon structure in both *Leptospirillum* species (Fig. 1) differs from the convergent organization of *rpfF* and *rpfCG* described for *Xanthomonas spp*., *Stenotrophomonas maltophila* and *Xylella fastidiosa*^32^. Consequently, regulatory mechanisms of this gene cluster may differ in leptospirilli, and further experiments are needed to evaluate DSF effects on mixed biofilm cultures containing *L. ferriphilum*^*T*^.

As mentioned above, the c-di-GMP second messenger is associated with the control of several phenotypes including EPS production and biofilm formation^27^. In particular, QS is related to c-di-GMP metabolism in some Gram-negative acidophiles and potentially represents an important mechanism regulating both process^20,24,26,30,31^. The *L.* *ferriphilum*^T^ genome contains ten genes annotated as encoding putative DGCs, 13 genes encoding proteins containing both DGC and PDE specific GGDEF and EAL domains, and two c-di-GMP-specific PDE^43^. Among these genes, eight are RpfR homologous, which could have the potential to perceive and regulate DSF effects, as described in *Burkholderia* and other *rpfR* containing species (Supplemental Fig. S1). Furthermore, four additional genes encoding HD/HDc domain-containing proteins and three genes encoding PilZ domain-containing c-di-GMP effector proteins were found. The latter genes were annotated as being related to functions such as cellulose and extracellular polysaccharide biosynthesis and export. This suggested that c-di-GMP metabolism also had an important function in the regulation of EPS production and biofilm formation in *L.* *ferriphilum*^T^. Interestingly, the *dosC* gene (LFTS_01845) encoding a diguanylate cyclase (GGDEF) domain-containing protein presented significantly increased transcripts levels in both DSF- and AHL-treated cells. DosC (direct oxygen sensing cyclase) is a globin-coupled heme-based oxygen sensor protein, displaying DGC activity in response to oxygen availability and regulating biofilm formation in an oxygen-dependent manner in *Escherichia coli*^59^. Some phenotypes related to its regulation include the production of poly-N-acetylglucosamine (PNAG) and cellulose in strains able to produce them and it leads to increased biofilm formation and decreased swimming in some motile strains^60^. The increased transcript counts of DosC also supported a DSF induced transition to sessile lifestyle in *L. ferriphilum*^T^.

The active growth of *Leptospirillum* cells in the presence of AHLs was in contrast to inhibition in iron oxidation and cell growth of *L. ferriphilum* after DSF and BDSF addition (Fig. 6). The detrimental effect of DSF/BDSF was reflected by decreased transcript counts for genes associated with basic cellular functions such as carbohydrate transport and metabolism, amino acid transport and metabolism, energy production and conversion, cell wall membrane biogenesis, inorganic ion transport and metabolism, and cell motility. Gene *ndhF* (LFTS_01821) that encodes NAD(P)H-quinone oxidoreductase subunit 5 (of NDH-1) had one of the most significant decreases in transcript counts. The NDH-1 complex shuttles electrons from NAD(P)H, via FMN and iron-sulfur (Fe-S) centers, to quinones in the respiratory chain^61^. The change in transcripts levels of this gene potentially reflected a lower energy production capacity, in agreement with inhibition in cell growth. In addition, several genes related to cell wall membrane biogenesis presented lower transcript counts including glycosyltransferase genes *wcfN* (LFTS_00540) and *wbdM* (LFTS_00541), *gfcE* gene (LFTS_00550) that encodes polysaccharide export outer membrane protein and *wcaJ* (LFTS_00551) that codes for a polyprenyl glycosylphosphotransferase. Gene *atoS* (LFTS_01830) also showed a decrease in transcript counts that codes for a membrane-associated kinase in the AtoS/AtoC two-component system regulating e.g. inhibition of ornithine decarboxylase (ODC), a key enzyme in polyamine synthesis^62^. Polyamines are implicated in many physiological functions such as DNA replication and repair, transcription, protein synthesis, and post-translational protein modifications and its inhibition retards or stops cell growth^63^. In addition, AtoS/AtoC also regulates chemotaxis and flagellar regulons^63^. While a decrease in *atoS* transcripts would involve alterations in several cellular processes, no changes in transcript levels were detected in other *ato* operon genes or *atoC* and this variation could not be correlated with polyamine biosynthesis or fatty acid degradation. Interestingly, for many of the proteins regulated by the DSF/Rpf system in *X. campestris*, their alteration in abundance was not associated with changes at transcript levels, suggesting that both post-transcriptional regulation and post-translational turnover may occur. Therefore, to confirm any DSF/BSDF or AHL effect over cellular process that may lead to inhibition in iron oxidation and/or cell growth of *L. ferriphilum* and other acidophiles, it is important to complement future transcriptomic studies with quantitative proteomics.

Finally, we conclude that the data supported the hypothesis that *Leptospirillum spp*. DSF production may be part of a niche defense strategy of established mineral-attached microcolonies. Furthermore, we propose that the finding of efficient inhibition of biological iron oxidation by DSF family compounds has potential for a possible application to prevent unwanted bioleaching.

## Material and methods

### Bacteria and growth conditions

The species used in the study were the Gram-negative iron- and sulfur-oxidizing bacteria *At. ferrooxidans* ATCC 53993, *At. ferridurans* ATCC 33020^T^, and *Acidiferrobacter* sp. SPIII/3 DSM 27195; Gram-negative iron-oxidizing bacteria *L. ferriphilum* DSM 14647^T^ and *L. ferrooxidans* DSM 2705^T^; and the Gram-positive iron- and sulfur-oxidizing bacterium *S. thermosulfidooxidans* DSM 9293^T^. Bacterial cells were grown in Mackintosh basal salt solution (Mac medium) pH 2.0^65^ with 72 mM iron(II) supplied as FeSO_4_·7H_2_O. For *S. thermosulfidooxidans*^T^, the medium was amended with 0.02% (wt/vol) yeast extract. Mac medium, ferrous sulfate (FeSO_4_·7H_2_O, 200 g/L, pH 1.2), and yeast extract were autoclaved separately at 121 °C for 20 min. Cultures were incubated under constant shaking at 140 rpm. The *Acidithiobacillus* strains as well as *Acidiferrobacter* sp. SPIII/3 DSM 27195 and *L. ferrooxidans*

^T^ were grown at 30 °C, *L.* *ferriphilum*^T^ at 37 °C, and *S. thermosulfidooxidans*^T^ at 45 °C. Cells were also cultivated using pyrite as energy source. Cultures of *L.* *ferriphilum*^T^ and *L.* *ferrooxidans*^T^ for bioleaching experiments and extraction of signal compounds were prepared as described for iron-cultures regarding agitation and growth temperature using 100 mL Mac medium pH 2.0 with 2% (wt/vol) pyrite grains (50–100 µm) as sole substrate. Cultures were prepared in triplicate and inoculated with 10^7^ cells/mL per species. Growth curves were followed by cell counting under light microscopy as previously described^14,15,20^.

### Pyrite preparation

Pyrite concentrate with a grain size of 50–100 μm was used (Baia Mare, Romania) that was treated as previously described^66^. Briefly, 100 g pyrite grains were boiled in 200 mL 6 M HCl for 30 min and then washed with deionized water until the pH was neutral. Afterward, grains were stirred twice in 100 mL acetone for 30 min to remove soluble sulfur compounds by discarding the solvent after the treatment. After evaporation of residual acetone, the washed pyrite was stored in a nitrogen atmosphere, and sterilized for 12 h at 125 °C.

### Iron oxidation activity tests

Early stationary phase iron(II)- and pyrite-grown cells were harvested at 7000 g for 10 min at room temperature, washed, and subsequently re-suspended in Mac medium pH 2.0. In case of pyrite-grown cells, a low speed centrifugation step (500 g, 1 min) was used to remove small pyrite particles. Afterwards, the cells were used for inoculation of triplicate 50 mL assays in 100 mL Erlenmeyer flasks with Mac medium at pH 2.0 amended with DSF ((Z)-11-methyl-2-dodecenoic acid, CAS 677354-23-3; Sigma) and BDSF ((Z)-2-dodecenoic acid, CAS 55928-65-9; Sigma). The cells were exposed to the signal molecules for 16 h without addition of an energy source and incubated with agitation at the specified growth temperature of the respective bacterial species. Afterwards, 32 mM iron(II)-ions were added and the assays were further incubated while quantifying iron(II)-ions using the phenanthroline method^64^ and planktonic cells using a phase contrast microscope and a Thoma chamber. The inoculum size was set at 10^7^ cells/mL for all experiments with combined DSF and BDSF at 0.1, 1, and 5 µM per compound along with a smaller inoculum of 4 × 10^6^ cells/mL *L. ferriphilum*^T^ for exposure to individual DSF and BDSF compounds at 1 and 5 µM. Control assays were prepared by addition of an equal volume of dimethyl sulfoxide (DMSO) that was used as solvent for the DSF and BDSF stock solutions.

For cultures treated with *L.* *ferriphilum*^T^ extracts (0.2% vol/vol), early stationary phase iron(II)-grown cells were harvested at 7000 g for 10 min at room temperature, washed, and subsequently re-suspended in Mac medium pH 1.6. The cells were used for inoculation of 20 mL assays in 100 mL Erlenmeyer flasks with Mac medium at pH 1.6 and 72 mM iron(II)-ions for *L.* *ferrooxidans*^T^ and *Acidiferrobacter* sp. SPIII/3 cultures or 100 mM iron(II)-ions for *L.* *ferriphilum*^T^. Control assays were prepared by addition of an equal volume of hexane or ethanol that were used as solvent for the *L.* *ferriphilum*^T^ extracts. Flasks were incubated with agitation at 120 rpm and periodically sampled in order to follow iron(II) oxidation and planktonic cell growth as mentioned above.

### Extraction of signaling compounds from batch pyrite cultures of *Leptospirillum* spp

Axenic pyrite cultures of *L.* *ferriphilum*^T^ and *L. ferrooxidans*^T^ were prepared in triplicate for each sampling time point and sacrificed for metabolite extraction from the liquid cell culture and the colonized pyrite by addition of 100 mL dichloromethane (p.a.) and stirring for 30 min at room temperature at 500 rpm. Erlenmeyer flasks, other glass materials used for handling the extracts, and magnetic stirring bars were cleaned in a laboratory dishwasher and subsequently rinsed with 6 M HCl, de-ionized water, and twice with dichloromethane to exclude contamination of metabolite extracts. The organic phase was recovered using a separation funnel, concentrated by rota-evaporation at 750 mbar and 35 °C to a volume of 2–4 mL, and transferred to brown glass vials with Teflon sealed screw caps using a glass Pasteur pipette and stored at -20 °C. Residual dichloromethane was evaporated prior to use of the extracts using a pure nitrogen stream before directly re-dissolving in 500 µL ethanol (p.a.). Solvents and chemicals were of analytical grade (p.a.) and supplied by VWR.

### Bioassay for BDSF/DSF

The bioassay was performed as previously described^41^. Briefly, *B. cenocepacia* H111–rpfF_*Bc*_ (pan-L15) was grown in Luria Bertani (LB) medium with chloramphenicol (80 µg/mL) and kanamycin (100 µg/mL) at 30 °C. A fresh overnight culture at an OD 600 of 1.8–2.0 was diluted 1:1 with LB medium from which, 200 μL was added to 96-well plates (transparent, Brandt)^41^. Before addition of the biosensor culture, synthetic standard compounds or extracts from acidophile bacterial cultures were added to the plate in triplicate. Synthetic BDSF, DSF, and various AHL standards dissolved in DMSO were added to individual wells in the 96-well plate (DMSO was used for the control). Extracts from *Leptospirillum* spp. pyrite cultures (300 µL) were re-dissolved in ethanol and added to individual wells in the plate. The solvent was evaporated at room temperature in a laminar flow chamber before addition of the biosensor culture. The plates were incubated for 20 h at 30 °C. Afterwards, 2 μL decanal (Sigma) was added to each well to detect luciferase activity of the biosensor using a luminometer. Luminescence levels in arbitrary units (AU) were recorded using a plate reader (FLUOstar Omega™, BMG Labtech®).

### Identification of DSF in culture extracts using GC–MS

A Thermo Scientific Trace 1310 GC and ISQ Single Quadrupole Mass Detector (ISQ-MS) operated in auto-sampler mode with helium as carrier gas and a non-polar capillary GC column was used (DB-5MS, 30 m × 0.25 µm, ID 0.25 mm, J&W Scientific, USA). Mass spectra were obtained by electron impact ionization (70 eV), the front inlet temperature was 250 °C, and injection was done in split less mode. The mass transfer line temperature was 250 °C and the temperature program was: initial temperature 50 °C (hold for 2 min) and raised to 260 °C with increasing rate of 15 °C/min and final temperature was hold for 5 min (total run time 21 min). DSF and BDSF were used as standards.

### RNA extraction, rRNA depletion, library preparation, and sequencing

Planktonic cells from iron(II) cultures of *L.* *ferriphilum*^T^ were grown to early stationary phase in Mac medium (pH 1.8, 37 °C) with 72 mM iron(II)-ions and harvested by centrifugation (7,000 g, 10 min). The cultures were inoculated at 10^8^ cells/mL in 100 mL Erlenmeyer flasks with 50 mL Mac medium (pH 1.8). Three cultivation conditions were compared by transcriptomic analyses. Namely, cultures were amended with combined (1) DSF and BDSF (2 µM each), (2) a mixture of AHLs namely N-dodecanoyl-DL-homoserine lactone (C12-AHL, CAS 8627-38-8, Sigma), N-tetradecanoyl-DL-homoserine lactone (C14-AHL, CAS 98206-80-5, Sigma), N-(3-hydroxydodecanoyl)-DL-homoserine (3-OH-C12-AHL, CAS 182359-60-0), and N-(3-hydroxytetradecanoyl)-DL-homoserine lactone (3-OH-C14-AHL, CAS 172670-99-4) at 5 µM per compound or (3) DMSO for the control assay. DMSO was used as solvent for signal compound stock solutions. After inoculation, 32 mM iron(II)-ions were added. The assay flasks were incubated for 2 h at 37 °C with agitation at 140 rpm and periodically sampled for determination of iron(II) concentrations using the phenanthroline method^65^. Within this time, the inhibitory effect of DSF/BDSF on iron(II) oxidation was confirmed and subsequently the flasks were rapidly cooled on ice and by addition of 1 volume ice-cold Mac medium (pH 1.8). The cultures (*n* = 4 per condition) were centrifuged at 12,500×*g* for 10 min at 4 °C. The resulting cell pellet was washed twice by re-suspending in 2 mL of sterile, ice-cold Mac medium (pH 1.8) and then flash frozen in liquid nitrogen and stored at − 80 °C.

Nucleic acid extraction was conducted as described^67^ with modifications. Briefly, the cells were re-suspended in lysis buffer (0.02% sodium acetate, 2% sodium dodecyl sulfate, 1 mM EDTA, pH 5.5) and lysed using Tri-reagent (Ambion) and the lysate was treated with bromo-chloro propane. Nucleic acids were precipitated with isopropanol, cleaned with 80% ethanol, dried at room temperature, and subsequently treated with DNAse I (Thermo Fisher Scientific®). Ribosomal RNA depletion was conducted using the QIAseq® FastSelect™-5S/16S/23S kit for bacterial RNA samples. Nucleic acid quantification and quality control were assessed by agarose gel electrophoresis, NanoDrop, Qubit™ RNA HS Assay kit (Invitrogen®), and the Agilent 2100 Bioanalyzer. Libraries (12 in total) were prepared by SciLifeLab, Stockholm, Sweden using the Illumina TruSeq stranded mRNA Kit. Paired-end sequencing (2 × 151 bp) was performed on one Illumina NovaSeq6000 lane using 'NovaSeqXp' workflow in 'S4' mode flowcell.

### Bioinformatics and statistics

The Bcl to FastQ conversion was performed using bcl2fastq_v2.20.0.422 from the CASAVA software suite. The quality scale used was Sanger/phred33/Illumina 1.8+ . at SciLifeLab, Stockholm, Sweden. The quality of the raw sequencing reads was assessed with FastQC/MultiQC^68,69^. Subsequently, adapter sequences were removed using Cutadapt/TrimGalore 0.6.1^70,71^. Paired-end transcript reads were mapped against the reference genome with the accession number GCA_900198525^38^ using Bowtie2^72^, sorted by their genomic location using the Samtools sort function, and counted using FeatureCounts of the Rsubreads package^73^. Raw counts were then processed for assessment of statistically significant differential gene expression with DESeq2^74^ by determination of LFC and corresponding *p* values (*p*adj, adjusted *p* values < 0.05). Differentially expressed genes (|LFC|> 1, *p*adj < 0.05) were analyzed using the clusters of orthologous groups (COG) database^75^.

## Supplementary Information

Supplementary Information.

## Data Availability

The raw sequencing data for the twelve axenic culture samples were deposited in ArrayExpress at the European Bioinformatics Institute under the accession E-MTAB-9845.
