# Supplementary material for: Diffusible signal factor signaling controls bioleaching activity and niche protection in the acidophilic, mineral-oxidizing leptospirilli
Source: Sci Rep. 2021 Aug 11;11:16275. doi: 10.1038/s41598-021-95324-9 (PMC8357829; doi:10.1038/s41598-021-95324-9)
Supplement: Supplementary file 1 — Supplementary Information. [file 41598_2021_95324_MOESM1_ESM.docx]

Supplemental Information for:

**Diffusible signal factor signaling controls bioleaching activity and niche protection
in the acidophilic, mineral-oxidizing Leptospirilli**

Sören Bellenberg^1,^*, Beatriz Salas^2^, Suresh Ganji^3^, Cristian Jorquera-Román^4^, Maria Luisa Valenzuela^5^, Antoine Buetti-Dinh^6,7^, C. Rikard Unelius^3^, Mark Dopson^1^, Mario Vera^2,4,^*


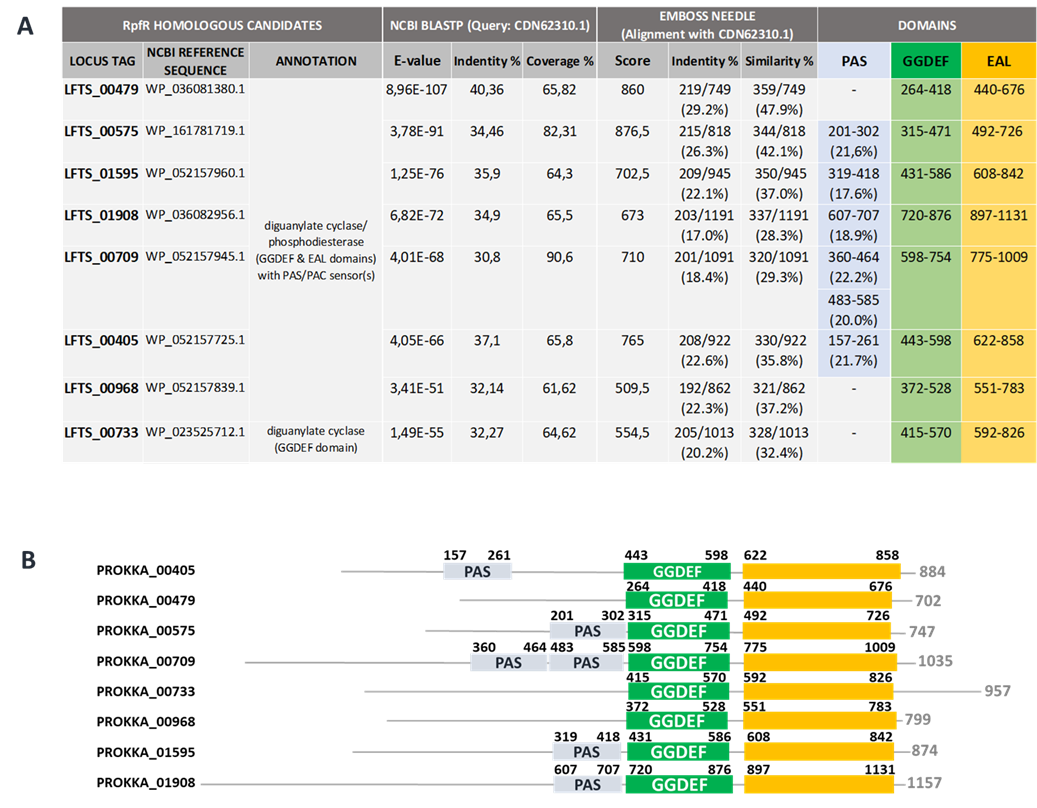


**Supplemental Fig. S1.** **RpfR homologous candidates in *L. ferriphilum*^T^ identified by bioinformatic analysis.** Results of bioinformatic search performed with a local BlastP analysis using amino acid sequence (NCBI accession N° CDN62310.1) as query and the CDSs of *L. ferriphilum* DSM 14647^T^ as reference genome (see https://doi.org/10.1128/AEM.02091-17). The identified sequences were further aligned with query using Emboss Needle tool to determinate their identity and similarity percent. The presence of PAS, GGDEF, and/or EAL domains was identified searching conserved domains in their NCBI reference sequences (CDD Search) (A). Organization of PAS, GGDEF, and/or EAL domains in genes encoding the RpfR homologous candidates in the genome of *L. ferriphilum*^T^ (B).

**Supplemental Fig. S2. Bioassay detection of synthetic commercially available BDSF and DSF after extraction with dichloromethane from acidic medium.** Data are averages ± SD of luminescence reads from triplicate experiments (*n* = 3).

**Supplemental Fig. S3. Bioassay detecting synthetic and commercially available BDSF and DSF.** The triangle indicates luminescence levels observed in control samples while diamonds and boxes indicate luminescence levels in assays with BDSF or DSF, respectively. Data are averages ± SD of luminescence reads from triplicate experiments (*n* = 3). Linear fittings and respective coefficients of determination (R^2^) are shown.

**
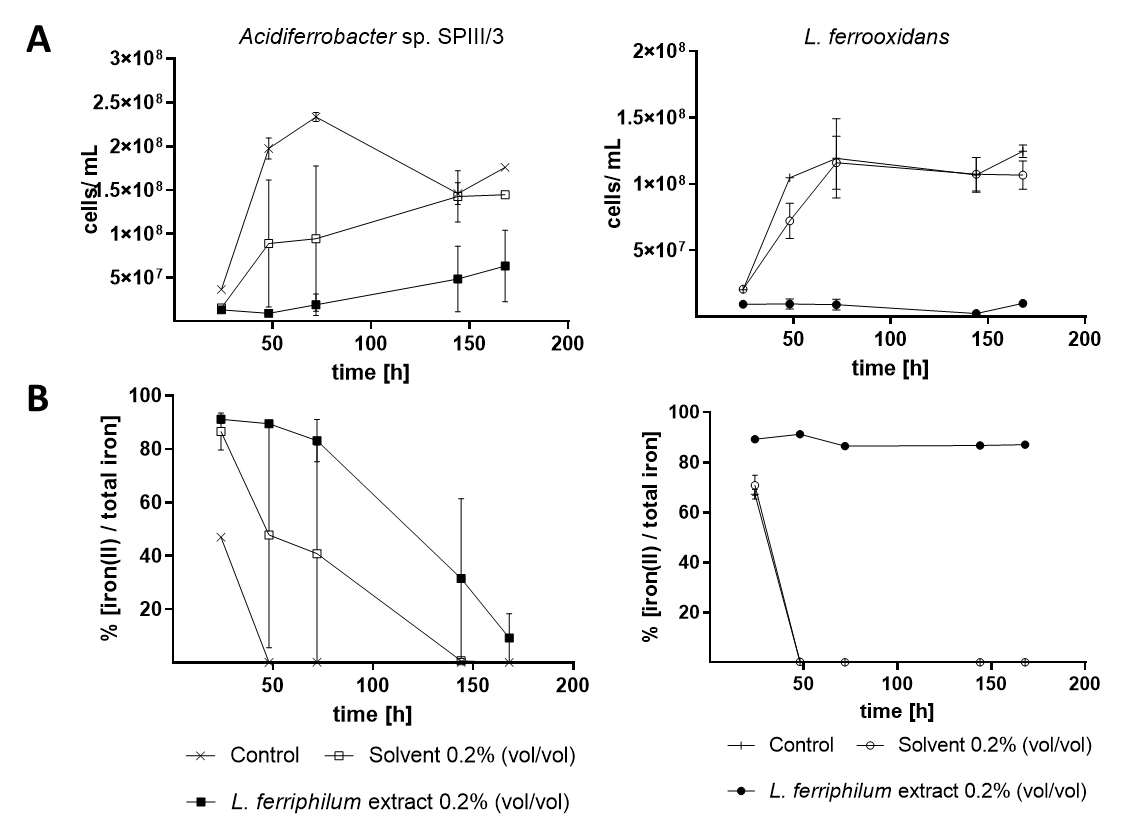
**

**Supplemental Fig. S4. *L. ferriphilum* DSM 14647^T^** **extracts inhibit *Acidiferrobacter* sp. SPIII/3 and *L. ferrooxidans*^T^ in iron oxidation and cell growth.** Cultures were inoculated with 1 × 10^7^ cells/mL cells of each iron- oxidizing bacteria and 4 g/L iron(II)-ions were added. Cells were exposed to *L. ferriphilum* DSM 14647^T^ extract 0.2 % (vol/vol) or solvent at the same concentration (hexane 0.2% vol/vol). Growth in the presence of *L. ferriphilum* DSM 14647^T^ extracts was affected as shown for iron(II)-grown cells (A). Iron(II)-grown cells were strongly inhibited in iron(II) oxidation (B). Data are averages ± SEM obtained from duplicate experiments (n = 2). No SEM bar appears for certain points when errors are shorter than the size of the symbol.


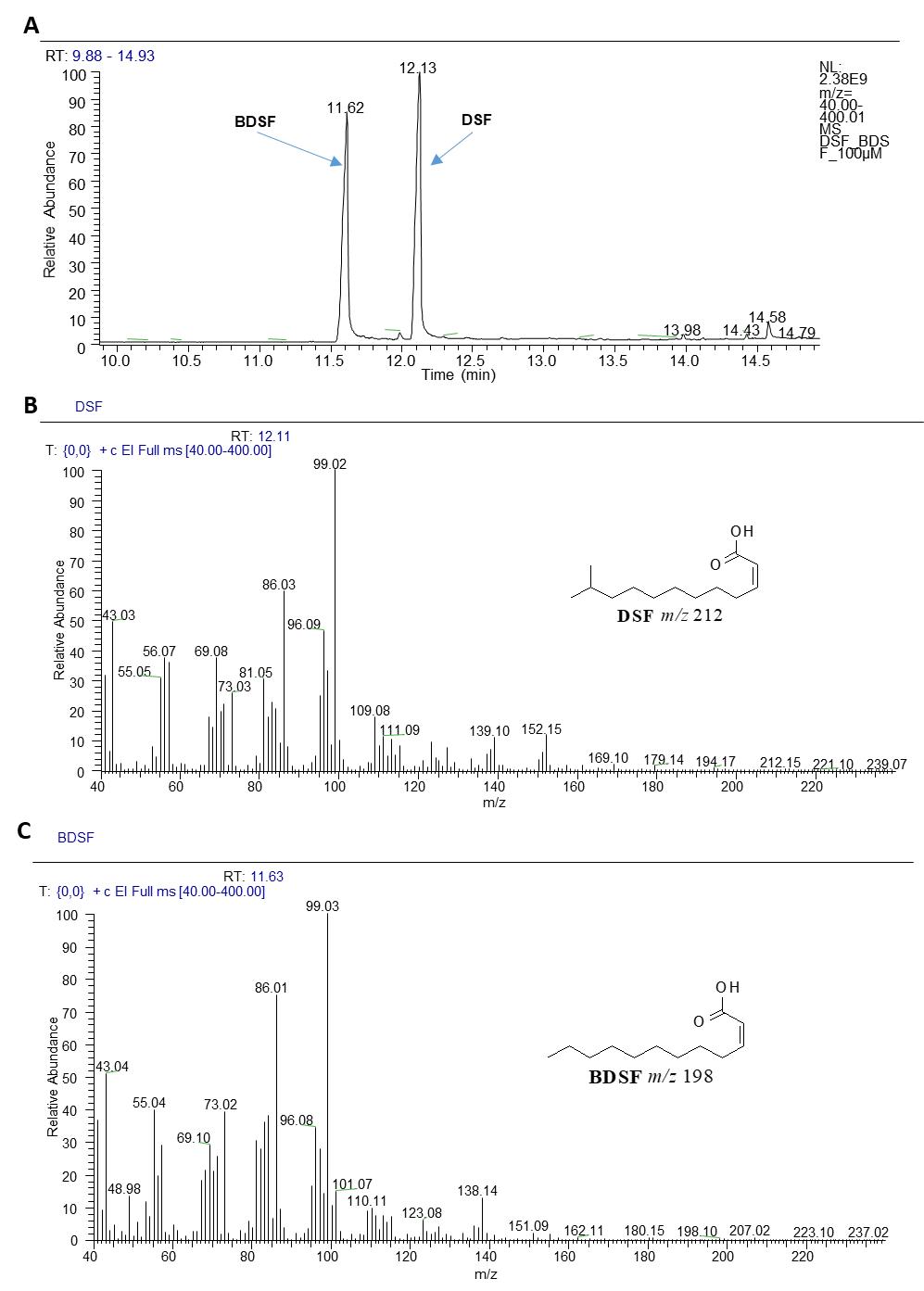


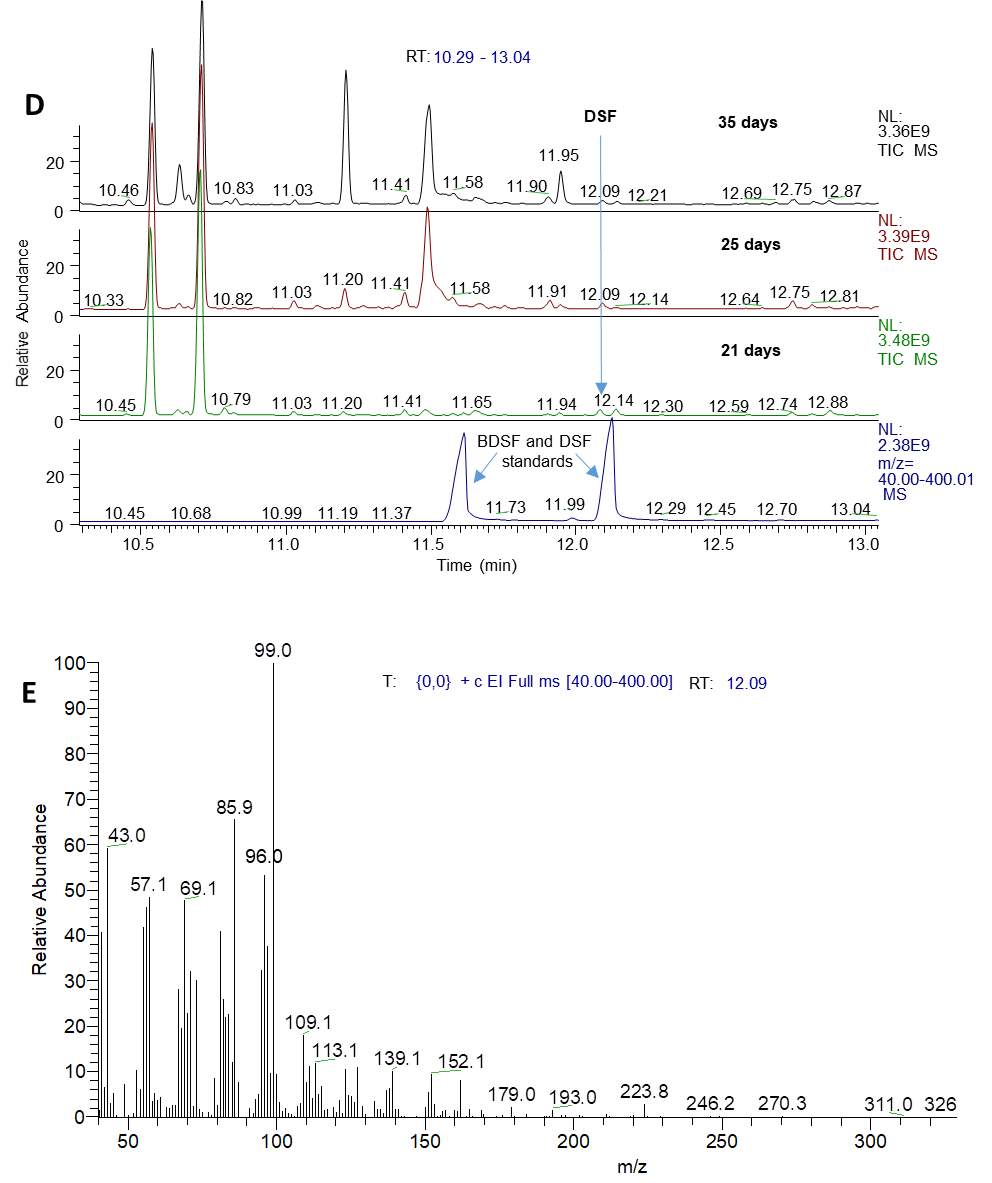


**
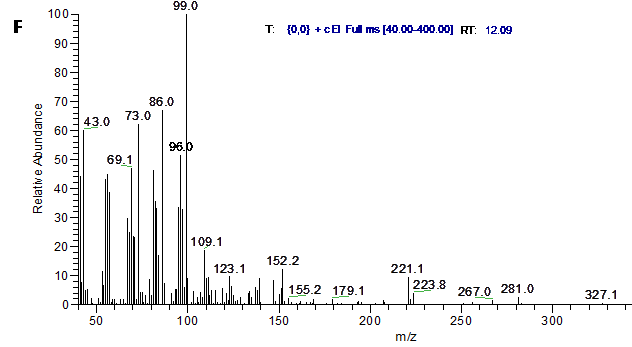
**

**Supplemental Fig. S5. GC-MS analysis of culture extracts for identification of biogenic DSF.** Synthetic DSF and BDSF were subjected to GC-MS analysis resulting in characteristic retention times shown in total ion chromatograms (A) plus EI fragmentation mass spectra of DSF (B) and BDSF (C). Total ion chromatograms of extracts from 21, 25, and 35 days old *L. ferrooxidans* DSM 2705 pyrite cultures measured by GC-MS are shown in (D). The mass spectrum at the inferred retention time of DSF (12.09 min) confirmed the presence of the biogenic substance in pyrite culture supernatants of *L. ferrooxidans* DSM 2705 (E) and *L. ferriphilum* DSM 14647^T^ (F).

**Supplemental Table S1.** **Differentially expressed genes in iron(II)-grown cells of *Leptospirillum ferriphilu*m DSM 14647.** Data are from cells after two hour exposure to 2 µM DSF and 2 µM BDSF (|LFC| > 1, padj < 0.05) determined by DESeq2 analysis of raw counts of RNASeq data from quadruplicate samples.

| locus tag | LFC | p value  (padJ) | gene name | annotation | NCBI Reference Sequence |
| --- | --- | --- | --- | --- | --- |
| LFTS_00939 | 1,74 | 1.40E-83 | rutR | transcriptional regulator TetR family | WP_036082305.1 |
| LFTS_00940 | 1,40 | 1.21E-24 | nrfB | Cytochrome c554 and c-prime | WP_036082306.1 |
| LFTS_00941 | 1,14 | 1.01E-14 | emrA | membrane fusion protein multidrug efflux system | WP_099590644.1 |
| LFTS_01634 | 1,27 | 2.59E-12 | otsB | trehalose 6-phosphate phosphatase | WP_081938200.1 |
| LFTS_01845 | 1,07 | 1.83E-08 | dosC | diguanylate cyclase (GGDEF) domain-containing protein | WP_049713715.1 |
| LFTS_02071 | 2,28 | 6.00E-25 | cusC | efflux transporter outer membrane factor (OMF) lipoprotein NodT family | WP_014961731.1 |
| LFTS_02072 | 1,14 | 7.36E-34 | acrB | multidrug efflux pump | WP_036081968.1 |
| LFTS_02073 | 1,01 | 5.52E-18 | acrA | membrane fusion protein multidrug efflux system | WP_099590670.1 |
| LFTS_00197 | -3,65 | 1.30E-05 | bamD | Tetratricopeptide repeat-containing protein | WP_036080014.1 |
| LFTS_00198 | -2,57 | 3.30E-03 | flrB | two-component system sensor histidine kinase FlrB | WP_023524390.1 |
| LFTS_00200 | -2,66 | 2.30E-04 | flgB | flagellar basal-body rod protein FlgB | WP_036080025.1 |
| LFTS_00201 | -2,47 | 8.41E-04 | flgC | flagellar basal-body rod protein FlgC | WP_036080028.1 |
| LFTS_00202 | -2,48 | 7.87E-05 | fliE | flagellar hook-basal body complex protein FliE | WP_036080031.1 |
| LFTS_00203 | -2,11 | 1.27E-03 | fliF | flagellar M-ring protein FliF | WP_036080033.1 |
| LFTS_00204 | -1,44 | 2.10E-02 | fliG | flagellar motor switch protein FliG | WP_036080037.1 |
| LFTS_00212 | -3,56 | 2.27E-05 | fliL | flagellar FliL protein | WP_014960005.1 |
| LFTS_00213 | -4,33 | 8.19E-06 | fliN/fliY | flagellar motor switch protein FliN/FliY | WP_014960006.1 |
| LFTS_00214 | -2,79 | 3.66E-04 | fliO/fliZ | flagellar protein FliO/FliZ | WP_023524379.1 |
| LFTS_00215 | -2,00 | 6.20E-03 | fliP | flagellar biosynthetic protein FliP | WP_023524378.1 |
| LFTS_00216 | -2,86 | 3.73E-05 | fliQ | flagellar biosynthetic protein FliQ | WP_042224543.1 |
| LFTS_00217 | -3,34 | 2.27E-05 | fliR | flagellar biosynthetic protein FliR | WP_023524376.1 |
| LFTS_00218 | -2,07 | 9.91E-03 | flhB | flagellar biosynthetic protein FlhB | WP_023524375.1 |
| LFTS_00219 | -2,10 | 3.30E-03 | flhA | flagellar biosynthesis protein FlhA | WP_023524374.1 |
| LFTS_00220 | -3,69 | 2.38E-05 | flhF | flagellar biosynthesis protein FlhF | WP_049713581.1 |
| LFTS_00221 | -3,08 | 5.65E-04 | flhF | flagellar biosynthesis protein FlhG | WP_036080049.1 |
| LFTS_00222 | -2,25 | 4.63E-03 | fliA | RNA polymerase sigma factor for flagellar operon FliA | WP_023524371.1 |
| LFTS_00266 | -1,69 | 1.08E-20 | pilF | Tetratricopeptide repeat-containing protein | WP_023524333.1 |
| LFTS_00267 | -2,67 | 1.55E-14 |  | hypothetical protein | WP_038506626.1 |
| LFTS_00268 | -3,03 | 1.78E-17 |  | hypothetical protein | WP_014960068.1 |
| LFTS_00418 | -1,21 | 6.63E-02 | hscB | Co-chaperone protein HscB | WP_052157754.1 |
| LFTS_00419 | -1,16 | 4.56E-02 | ycf57 | iron-sulfur cluster assembly protein | WP_014960239.1 |
| LFTS_00420 | -1,58 | 6.87E-03 | iscU | nitrogen fixation protein NifU | WP_036081256.1 |
| LFTS_00421 | -1,85 | 4.67E-03 | icsS2 | cysteine desulfurase | WP_036081258.1 |
| LFTS_00422 | -1,25 | 2.50E-02 |  | transcriptional regulator BadM/Rrf2 family | WP_099590678.1 |
| LFTS_00439 | -1,26 | 1.47E-03 | cydA | cytochrome d ubiquinol oxidase subunit I | WP_036081291.1 |
| LFTS_00455 | -1,14 | 3.75E-08 |  | hypothetical protein | WP_143469079.1 |
| LFTS_00458 | -1,48 | 2.34E-04 |  | hypothetical protein | WP_036081346.1 |
| LFTS_00502 | -1,68 | 2.34E-04 | napB | PEP-CTERM protein-sorting domain-containing protein | WP_143469085.1 |
| LFTS_00518 | -1,22 | 3.38E-06 |  | PEP-CTERM protein-sorting domain-containing protein | WP_052157770.1 |
| LFTS_00540 | -1,31 | 7.89E-03 | wcfN | Glycosyltransferase GT2 family | WP_036081527.1 |
| LFTS_00541 | -1,86 | 1.55E-03 | wbdM | Glycosyltransferase involved in cell wall biosynthesis | WP_052157775.1 |
| LFTS_00550 | -1,24 | 1.16E-03 | gfcE | polysaccharide export outer membrane protein | WP_036081550.1 |
| LFTS_00551 | -1,07 | 2.30E-05 | wcaJ | sugar transferase PEP-CTERM system associated/exopolysaccharide biosynthesis polyprenyl glycosylphosphotransferase | WP_036081553.1 |
| LFTS_00566 | -1,46 | 1.98E-02 | wcaG | UDP-glucuronate 4-epimerase | WP_036081600.1 |
| LFTS_00569 | -2,11 | 3.36E-03 | glmS | glucosamine--fructose-6-phosphate aminotransferase (isomerizing) | WP_036081614.1 |
| LFTS_00576 | -1,18 | 3.52E-07 |  | PEP-CTERM protein-sorting domain-containing protein | WP_036081628.1 |
| LFTS_00581 | -1,08 | 9.56E-06 |  | aconitase | WP_036081638.1 |
| LFTS_00642 | -1,11 | 3.40E-03 |  | hypothetical protein | WP_143469103.1 |
| LFTS_00645 | -1,18 | 7.82E-17 | sahH | adenosylhomocysteinase | WP_036081726.1 |
| LFTS_00646 | -1,23 | 4.97E-13 | thiS | sulfur carrier protein | WP_036081727.1 |
| LFTS_00966 | -1,02 | 1.95E-06 |  | putative isocitrate dehydrogenase (NADP) | WP_099590683.1 |
| LFTS_01160 | -1,51 | 1.72E-03 |  | Molybdate transporter of MFS superfamily protein | WP_180271586.1 |
| LFTS_01186 | -2,54 | 4.90E-04 | nasT | response regulator receiver and ANTAR domain protein | WP_099590487.1 |
| LFTS_01187 | -2,36 | 3.02E-05 | pilJ | Rop-like protein | WP_099590488.1 |
| LFTS_01188 | -1,71 | 1.83E-02 | nifV | homocitrate synthase NifV | WP_099590489.1 |
| LFTS_01189 | -1,13 | 4.08E-02 | thiF | Molybdopterin or thiamine biosynthesis adenylyltransferase | WP_180271596.1 |
| LFTS_01202 | -2,33 | 1.88E-06 | nasA | MFS transporter NNP family nitrate/nitrite transporter | WP_099590501.1 |
| LFTS_01203 | -2,81 | 1.01E-12 | glnB | nitrogen regulatory protein P-II family protein | WP_099590502.1 |
| LFTS_01204 | -2,64 | 1.03E-07 | yedY | sulfoxide reductase catalytic subunit YedY | WP_180271610.1 |
| LFTS_01205 | -1,58 | 6.97E-08 |  | hypothetical protein | WP_099590657.1 |
| LFTS_01217 | -1,61 | 1.53E-02 | ilvD | dihydroxyacid dehydratase | WP_099590514.1 |
| LFTS_01218 | -3,10 | 6.23E-05 |  | hypothetical protein | WP_099590515.1 |
| LFTS_01219 | -4,55 | 7.36E-10 |  | hypothetical protein | WP_099590516.1 |
| LFTS_01220 | -5,23 | 3.63E-18 | ndhF | NAD(P)H-quinone oxidoreductase subunit 5 | WP_099590517.1 |
| LFTS_01429 | -1,28 | 2.55E-03 | uspA | Nucleotide-binding universal stress protein UspA family | WP_036082839.1 |
| LFTS_01444 | -1,62 | 4.83E-27 | ccmB | Cytochrome C biogenesis protein transmembrane region | WP_036082848.1 |
| LFTS_01445 | -1,65 | 2.10E-04 |  | Methyl-accepting chemotaxis protein | WP_036082849.1 |
| LFTS_01451 | -1,17 | 1.04E-03 | ndhF | NADH-quinone oxidoreductase subunit F | WP_036083835.1 |
| LFTS_01463 | -2,82 | 3.66E-05 | virB2 | TrbC/VIRB2 family protein | WP_036083818.1 |
| LFTS_01464 | -2,83 | 4.87E-05 |  | hypothetical protein | WP_036083816.1 |
| LFTS_01465 | -2,19 | 4.07E-03 | traC | Type IV secretory pathway VirB4 component | WP_036083814.1 |
| LFTS_01519 | -2,10 | 1.72E-04 | guaA | GMP synthase (glutamine-hydrolysing) | WP_036084152.1 |
| LFTS_01598 | -1,26 | 9.84E-04 |  | hypothetical protein | WP_158423151.1 |
| LFTS_01599 | -1,18 | 3.07E-02 |  | hypothetical protein | WP_014961248.1 |
| LFTS_01734 | -2,10 | 6.46E-04 | acfB | Cache domain-containing protein | WP_014961415.1 |
| LFTS_01746 | -1,16 | 3.69E-02 |  | circularly permuted type 2 ATP-grasp protein | WP_036083733.1 |
| LFTS_01821 | -7,28 | 2.06E-24 | ndhF | NAD(P)H-quinone oxidoreductase subunit 5 | WP_099590663.1 |
| LFTS_01822 | -6,69 | 8.40E-15 |  | hypothetical protein | WP_036082909.1 |
| LFTS_01823 | -5,06 | 1.61E-06 |  | hypothetical protein | WP_038507267.1 |
| LFTS_01824 | -3,24 | 5.10E-04 | ilvD | dihydroxyacid dehydratase | WP_036082910.1 |
| LFTS_01828 | -4,92 | 9.91E-17 | ndhF | NAD(P)H-quinone oxidoreductase subunit 5 | WP_052157928.1 |
| LFTS_01829 | -4,38 | 1.02E-09 |  | hypothetical protein | WP_036082912.1 |
| LFTS_01830 | -1,46 | 9.71E-03 | atoS | PAS domain S-box-containing protein | WP_052157935.1 |
| LFTS_02089 | -1,91 | 8.41E-03 |  | hypothetical protein | WP_036081993.1 |
| LFTS_02090 | -1,82 | 6.32E-03 |  | Peptidase C39 family protein | WP_143469023.1 |
| LFTS_02091 | -3,92 | 1.72E-06 |  | hypothetical protein | WP_014961752.1 |
| LFTS_02092 | -4,67 | 3.27E-08 |  | hypothetical protein | WP_014961753.1 |
| LFTS_02112 | -1,17 | 6.35E-02 |  | hypothetical protein | KGA94065.1 |
| LFTS_02293 | -1,41 | 9.83E-11 |  | hypothetical protein | WP_038506256.1 |
| LFTS_02331 | -2,20 | 4.30E-04 |  | Glycosyltransferase involved in cell wall bisynthesis | WP_052157734.1 |
| LFTS_02332 | -3,42 | 1.36E-05 | cgeB | Glycosyl transferases group 1 | WP_036080682.1 |
| LFTS_02345 | -1,01 | 6.53E-03 | flgI | flagellar P-ring protein precursor FlgI | WP_036080716.1 |
| LFTS_02346 | -1,73 | 1.10E-03 | flgH | flagellar L-ring protein precursor FlgH | WP_036080719.1 |
| LFTS_02347 | -1,78 | 1.19E-02 | flgA | flagella basal body P-ring formation protein FlgA | WP_036080722.1 |
| LFTS_02348 | -1,92 | 3.30E-03 | flgG | flagellar basal-body rod protein FlgG | WP_036080725.1 |
| LFTS_02349 | -3,97 | 2.27E-05 | flgE | flagellar basal-body rod protein FlgF | WP_036080728.1 |
| LFTS_02488 | -1,28 | 1.49E-02 | lpxC | UDP-3-O-[3-hydroxymyristoyl] N-acetylglucosamine deacetylase | WP_036081051.1 |
| LFTS_02527 | -2,27 | 2.33E-24 | mshN | TPR repeat-containing protein | WP_036083913.1 |
| LFTS_02528 | -1,96 | 1.03E-28 |  | Putative MetA-pathway of phenol degradation | WP_036083915.1 |
| LFTS_02529 | -1,04 | 1.72E-15 |  | hypothetical protein | WP_036083917.1 |

**Supplemental Table S2.** **Differentially expressed genes (|LFC| > 2, padj < 0.005) in iron(II)-grown cells of *Leptospirillum ferriphilu*m DSM 14647.** Data are from cells after two hour exposure to AHLs (C12-AHL, C14-AHL, OH-C12-AHL, OH-C14-AHL; 2 µM each) determined by DESeq2 analysis of raw counts of RNASeq data from quadruplicate samples.

| Locus tag | **LFC** | **p value**  **(padj)** | **Gene name** | **Annotation** | **NCBI Reference Sequence** |
| --- | --- | --- | --- | --- | --- |
|  |  |  |  |  |  |
| LFTS_00021 | 2.01 | 3.57E-87 |  | ChrR Cupin-like domain-containing protein | WP_036079647.1 |
| LFTS_00030 | 2.03 | 2.40E-193 |  | carbonic anhydrase | WP_036079668.1 |
| LFTS_00298 | 2.85 | 0.00E+00 |  | phosphoribosylformylglycinamidine synthase | WP_023524261.1 |
| LFTS_00311 | 2.30 | 1.37E-56 | glnB | nitrogen regulatory protein P-II family protein | WP_023524249.1 |
| LFTS_00312 | 2.37 | 1.04E-64 | amt-1 | ammonium transporter (TC 1.A.11) | WP_014960129.1 |
| LFTS_00324 | 5.26 | 8.62E-07 | arsR | ArsR family transcriptional regulator | WP_101494939.1 |
| LFTS_00325 | 4.23 | 4.11E-05 | tolC | Outer membrane protein TolC | WP_036080151.1 |
| LFTS_00326 | 3.33 | 7.31E-04 | czcB | RND family efflux transporter MFP subunit | WP_023524235.1 |
| LFTS_00327 | 2.85 | 7.31E-04 | acrB | Multidrug efflux pump subunit AcrB | WP_023524234.1 |
| LFTS_00329 | 2.05 | 1.12E-03 | adhN | alcohol dehydrogenase propanol-preferring | WP_020859449.1 |
| LFTS_00413 | 2.08 | 3.81E-145 | cbiA | ATP-binding protein involved in chromosome partitioning | WP_036081242.1 |
| LFTS_00415 | 2.36 | 1.55E-56 | iscX | FeS assembly protein IscX | WP_036081244.1 |
| LFTS_00416 | 2.22 | 9.70E-46 | fdx | ferredoxin 2Fe-2S | WP_036081247.1 |
| LFTS_00417 | 2.30 | 1.22E-10 | hscA | molecular chaperone HscA | WP_036081250.1 |
| LFTS_00418 | 2.51 | 3.52E-07 | hscB | Co-chaperone protein HscB | WP_052157754.1 |
| LFTS_00419 | 2.24 | 1.00E-06 | iscA | iron-sulfur cluster assembly protein | WP_014960239.1 |
| LFTS_00420 | 2.23 | 5.48E-06 | iscU | nitrogen fixation protein NifU | WP_036081256.1 |
| LFTS_00421 | 2.15 | 1.61E-04 | icsS2 | cysteine desulfurase | WP_036081258.1 |
| LFTS_00422 | 2.65 | 2.59E-10 | iscR | transcriptional regulator BadM/Rrf2 family | WP_099590678.1 |
| LFTS_00514 | 2.08 | 1.95E-55 | rpfF | DSF synthase | WP_036081469.1 |
| LFTS_00939 | 3.90 | 0.00E+00 | rutR | transcriptional regulator TetR family | WP_036082305.1 |
| LFTS_00940 | 3.28 | 1.30E-218 | nrfB | Cytochrome c554 and c-prime | WP_036082306.1 |
| LFTS_00941 | 2.84 | 6.03E-137 | emrA | membrane fusion protein multidrug efflux system | WP_099590644.1 |
| LFTS_00942 | 2.22 | 5.12E-124 | emrB | MFS transporter DHA2 family multidrug resistance protein | WP_036082308.1 |
| LFTS_00943 | 2.07 | 6.05E-197 | tolC | outer membrane protein | WP_052157836.1 |
| LFTS_01263 | 3.12 | 1.30E-250 |  | IPT/TIG domain-containing protein | WP_052157880.1 |
| LFTS_01557 | 2.71 | 1.19E-168 |  | DNA binding domain-containing protein excisionase family | WP_036083271.1 |
| LFTS_01631 | 4.15 | 1.05E-180 |  | putative arabinose efflux permease MFS family | WP_036083394.1 |
| LFTS_01632 | 3.10 | 1.14E-86 | glgA4 | Glycosyltransferase involved in cell wall bisynthesis | WP_036083397.1 |
| LFTS_01633 | 3.03 | 0.00E+00 | otsA | trehalose 6-phosphate synthase | WP_036083399.1 |
| LFTS_01709 | 2.50 | 1.78E-135 | lpoB | TolB amino-terminal domain-containing protein | WP_099590563.1 |
| LFTS_01710 | 3.46 | 0.00E+00 | mshN | Tetratricopeptide repeat protein | WP_099590564.1 |
| LFTS_01740 | 2.70 | 1.68E-76 | mshN | Tetratricopeptide repeat-containing protein | WP_036083683.1 |
| LFTS_01837 | 2.20 | 1.84E-77 |  | DinB family protein | WP_023525132.1 |
| LFTS_01838 | 2.21 | 5.44E-20 |  | 4-carboxymuconolactone decarboxylase | WP_042224350.1 |
| LFTS_01841 | 3.62 | 3.72E-186 | mshN | Tetratricopeptide repeat-containing protein | WP_023525129.1 |
| LFTS_01845 | 4.42 | 0.00E+00 | dosC | diguanylate cyclase (GGDEF) domain-containing protein | WP_049713715.1 |
| LFTS_01846 | 4.31 | 0.00E+00 | cheR | PAS fold-containing protein | WP_023525129.1 |
| LFTS_01912 | 2.43 | 4.18E-66 | erpA | iron-sulfur cluster insertion protein | WP_014961608.1 |
| LFTS_02016 | 2.05 | 5.27E-61 | pir | Pirin | WP_014961713.1 |
| LFTS_02071 | 5.59 | 0.00E+00 | cusC | efflux transporter outer membrane factor (OMF) lipoprotein NodT family | WP_014961731.1 |
| LFTS_02072 | 4.53 | 0.00E+00 | acrB | multidrug efflux pump | WP_036081968.1 |
| LFTS_02073 | 4.48 | 0.00E+00 | acrA | membrane fusion protein multidrug efflux system | WP_099590670.1 |
| LFTS_02094 | 2.00 | 2.03E-173 | ccoP | Cytochrome C oxidase cbb3-type subunit III | WP_014961757.1 |
| LFTS_02249 | 2.91 | 5.78E-296 | hsp | HSP20 family protein | WP_023524701.1 |
| LFTS_00197 | -3.66 | 1.86E-06 | bamD | Tetratricopeptide repeat-containing protein | WP_036080014.1 |
| LFTS_00198 | -2.61 | 8.70E-04 | flrB | two-component system sensor histidine kinase FlrB | WP_023524390.1 |
| LFTS_00200 | -2.64 | 4.43E-05 | flgB | flagellar basal-body rod protein FlgB | WP_036080025.1 |
| LFTS_00201 | -3.00 | 6.88E-06 | flgC | flagellar basal-body rod protein FlgC | WP_036080028.1 |
| LFTS_00202 | -2.66 | 8.33E-06 | fliE | flagellar hook-basal body complex protein FliE | WP_036080031.1 |
| LFTS_00203 | -2.23 | 1.03E-04 | fliF | flagellar M-ring protein FliF | WP_036080033.1 |
| LFTS_00212 | -3.16 | 4.02E-05 | fliL | flagellar FliL protein | WP_014960005.1 |
| LFTS_00213 | -3.91 | 5.02E-06 | fliN/fliY | flagellar motor switch protein FliN/FliY | WP_014960006.1 |
| LFTS_00214 | -3.19 | 1.86E-05 | fliO/fliZ | flagellar protein FliO/FliZ | WP_023524379.1 |
| LFTS_00216 | -2.94 | 4.62E-05 | fliQ | flagellar biosynthetic protein FliQ | WP_042224543.1 |
| LFTS_00217 | -3.54 | 8.25E-07 | fliR | flagellar biosynthetic protein FliR | WP_023524376.1 |
| LFTS_00218 | -2.22 | 9.21E-04 | flhB | flagellar biosynthetic protein FlhB | WP_023524375.1 |
| LFTS_00219 | -2.40 | 2.14E-04 | flhA | flagellar biosynthesis protein FlhA | WP_023524374.1 |
| LFTS_00220 | -3.29 | 2.71E-05 | flhF | flagellar biosynthesis protein FlhF | WP_049713581.1 |
| LFTS_00221 | -2.99 | 1.75E-04 | flgH | flagellar biosynthesis protein FlhG | WP_036080049.1 |
| LFTS_00267 | -2.01 | 1.32E-09 |  | Putative MetA-pathway of phenol degradation | WP_038506626.1 |
| LFTS_01186 | -2.82 | 1.82E-05 | nasT | response regulator receiver and ANTAR domain protein | WP_099590487.1 |
| LFTS_01187 | -2.13 | 6.24E-05 | pilJ | Rop-like protein | WP_099590488.1 |
| LFTS_01188 | -2.05 | 1.30E-03 | nifV | homocitrate synthase NifV | WP_099590489.1 |
| LFTS_01203 | -2.48 | 4.12E-11 | glnB-3 | nitrogen regulatory protein P-II family protein | WP_099590502.1 |
| LFTS_01204 | -2.11 | 3.27E-06 | yedY | sulfoxide reductase catalytic subunit YedY | WP_180271610.1 |
| LFTS_01220 | -5.06 | 1.16E-19 | ndhF | NAD(P)H-quinone oxidoreductase subunit 5 | WP_099590517.1 |
| LFTS_01465 | -2.31 | 7.34E-04 | traC | Type IV secretory pathway VirB4 component | WP_036083814.1 |
| LFTS_01519 | -2.39 | 1.47E-06 | guaA | GMP synthase (glutamine-hydrolysing) | WP_036084152.1 |
| LFTS_01547 | -2.99 | 2.91E-194 | acsF | acyl-[acyl-carrier-protein] desaturase | WP_036083261.1 |
| LFTS_01548 | -2.15 | 4.41E-30 |  | methyl-accepting chemotaxis protein | WP_036083266.1 |
| LFTS_01770 | -2.05 | 1.52E-40 |  | HDIG domain-containing protein | WP_014961466.1 |
| LFTS_01821 | -7.18 | 3.15E-25 | ndhF | NAD(P)H-quinone oxidoreductase subunit 5 | WP_099590663.1 |
| LFTS_01824 | -3.69 | 7.81E-06 | ilvD-1_2 | dihydroxyacid dehydratase | WP_036082910.1 |
| LFTS_01828 | -3.94 | 7.00E-13 | ndhF | NAD(P)H-quinone oxidoreductase subunit 5 | WP_052157928.1 |
| LFTS_01830 | -2.04 | 1.42E-05 | atoS | PAS domain S-box-containing protein | WP_052157935.1 |
| LFTS_01906 | -2.33 | 3.06E-226 | aspS | aspartyl-tRNA synthetase | WP_036082954.1 |
| LFTS_02287 | -2.20 | 2.00E-67 | pstA | phosphate transport system permease protein | WP_036080603.1 |
| LFTS_02332 | -3.27 | 7.24E-06 | cgeB | Glycosyl transferases group 1 | WP_036080682.1 |
| LFTS_02348 | -2.43 | 2.29E-05 | flgG | flagellar basal-body rod protein FlgG | WP_036080725.1 |
| LFTS_02349 | -3.98 | 3.63E-06 | flgE | flagellar basal-body rod protein FlgG | WP_036080728.1 |
